# Innovative Approach for Human Semen Quality Assessment Based on Volatilomics

**DOI:** 10.3390/toxics12080543

**Published:** 2024-07-27

**Authors:** Simonetta Capone, Angiola Forleo, Antonio Vincenzo Radogna, Valentina Longo, Giulia My, Alessandra Genga, Alessandra Ferramosca, Giuseppe Grassi, Flavio Casino, Pietro Siciliano, Tiziana Notari, Sebastiana Pappalardo, Marina Piscopo, Luigi Montano

**Affiliations:** 1 National Research Council, Institute for Microelectronics and Microsystems (CNR-IMM), 73100 Lecce, Italy; angiola.forleo@cnr.it (A.F.); antonio.radogna@unisalento.it (A.V.R.); valentinalongo2110@gmail.com (V.L.); my.giulia@imm.cnr.it (G.M.); flavio.casino@cnr.it (F.C.); pietroaleardo.siciliano@cnr.it (P.S.); 2 Department of Experimental Medicine, University of Salento, 73100 Lecce, Italy; alessandra.ferramosca@unisalento.it; 3 Department of Biological and Environmental Sciences and Technologies, University of Salento, 73100 Lecce, Italy; alessandra.genga@unisalento.it; 4 Department of Engineering for Innovation, University of Salento, 73100 Lecce, Italy; giuseppe.grassi@unisalento.it; 5 Reproductive Medicine Unit of Check Up Polydiagnostic Center, 84131 Salerno, Italy; tiziananotari7@gmail.com; 6 Reproduction and Fertility Center, 00128 Rome, Italy; pappalardo@riproduzionefertilita.it; 7 Department of Biology, University of Naples Federico II, 80138 Naples, Italy; marina.piscopo@unina.it; 8 Andrology Unit and Service of Lifestyle Medicine in UroAndrology, “S. Francesco d’Assisi” Hospital, Oliveto Citra, 84020 Salerno, Italy; 9 Coordination Unit of the Network for Environmental and Reproductive Health (EcoFoodFertility Project), “S. Francesco d’Assisi” Hospital, Oliveto Citra, 84020 Salerno, Italy; 10 Department of Biology, Tor Vergata University of Rome, 00133 Rome, Italy

**Keywords:** semen quality, human exposome, volatilomics, GC/MS

## Abstract

The volatilome profile of some biofluids (blood, urine, and human semen) identified by Solid-Phase Microextraction–Gas Chromatography/Mass Spectrometry (SPME-GC/MS) and collected from young men living in two high-pollution areas in Italy, i.e., Land of Fires and Valley of Sacco River, have been coupled to sperm parameters obtained by spermiogram analysis to build general multiple regression models. Panels of volatile organic compounds (VOCs) have been selected to optimize the models and used as predictive variables to estimate the different sperm quality parameters (sperm cell concentration, total and progressive motility/immotile cells, total/head/neck/tail morphology anomalies, semen round cell concentration). The results of the multiple linear regression models based on the different subgroups of data joining VOCs from one/two or three biofluids have been compared. Surprisingly, the models based on blood and urine VOCs have allowed an excellent estimate of spermiogram values, paving the way towards a new method of indirect evaluation of semen quality and preventive screening. The significance of VOCs in terms of toxicity and dangerousness was discussed with the support of chemical databases available online.

## 1. Introduction

Over the last several decades, a hotly debated topic is the meaningful decline of male infertility worldwide [1]. In a meta-analysis, Sengupta P et al. concisely presented evidence of decreased sperm concentration in European males over the past 50 years [2]. Recently, the findings put forth by Levine H et al. [3,4] with a prominent upgraded systematic review and meta-regression analysis reflected that sperm count, as well as other sperm quality parameters, are declining in men not only from North America, Europe, and Australia but also from South/Central America, Africa, and Asia; the warning is that human semen is globally deteriorating at an accelerate pace, constituting a significant public health problem. The contention is not universally accepted and is motivated by criticisms and limitations found in the reported studies. However, it must be emphasized that the moot point is not the deterioration of human semen quality but rather that this does not necessarily compromise the ability to conceive; hence, it does not automatically translate into a reduction in male fertility [5].

While it is difficult to determine the actual temporal trend in human semen quality in different geographical areas, it is crucial to understand the real impact of non-pathological factors, mainly environmental exposure, and lifestyle factors, on male fertility. The most correct approach to studying how multiple risk factors can undermine the quality of male semen is the human exposome. This is a new paradigm to encompass the totality of human environmental (meaning all non-genetic) exposures from conception onwards [6,7]. Numerous studies report different environmental (air, land, water pollution) and lifestyle factors (nutrients, tobacco, alcohol, and drug use; psychological stress; obesity; insufficient sleep; heat; etc.) that can affect semen quality [8]. However, some studies call for greater rigor in stating statistically significant associations between environmental pollutants and sperm parameters since the population recruited in this study and the methods of assessment of both pollution and semen quality parameters limit the generalizability of the findings [9]. Environment factors include pollution coming from several sources, such as motor vehicle exhaust, factories, fire, household, agriculture, waste treatment, oil refineries, and natural sources; pollution involves particulate matter, volatile organic compounds (VOCs), ozone, nitrogen oxides, sulfur dioxide, carbon monoxide, polycyclic aromatic hydrocarbons (PAHs), and radiations [10]. Agricultural and industrial activities have spread all over the world and are practiced more and more intensively, putting strong and constant pressure on the natural balance of ecosystems, which inevitably become polluted. In addition, the repeated disrespect for environmental protection measures and illegal waste management with the culpability of organized criminals greatly increase the health risk of the human population [11].

In ambient pollution, volatile organic compounds (VOCs) play a relevant role due to their potential hazard character. VOCs are chemicals with a high vapor pressure at room temperature and a standard atmospheric pressure, and they are generally classified by their volatility. VOCs can be divided between biological (endogenous or metabolite VOCs) and anthropogenic sources (exogenous VOCs). The latter are pervasive in daily life and are used in day-to-day activities, including industries, agriculture, office, home, transportation, etc. Though volatile compounds have an indispensable function in manufacturing and maintaining the stability of many products, the health impacts associated with their prolonged exposure cannot be overlooked [12,13]. VOCs are metabolized quickly and yield several toxic metabolites that are excreted in body biofluids. VOCs have, hence, aroused considerable scientific interest in human biomonitoring for studies of exposure to environmental contaminants [14]. Human volatilome, encompassing all the volatile organic compounds (VOCs) found in the human body (the volatilome), together with the related omics science (Volatilomics), are fundamental tools in evaluating the environmental and human health impacts of VOCs [15,16,17,18].

A particular interest is devoted to studying how exposure to low doses of VOCs and other contaminants (such as heavy metals, dioxins, plastic contaminants (bisphenols), pesticides and herbicides, phthalates) have adverse effects on reproductive health, mainly semen quality and male fertility [19,20,21]. Many of them accumulate in the organism, have negative synergic effects, and are endocrine-disrupting environmental pollutants, leading to adverse impacts on semen quality [22,23]. In both epidemiological and experimental studies, it was found that exposure to all these environmental contaminants led to alterations in sperm morphology, sperm motility, sperm count, protamine/histone ratios, sperm nuclear basic proteins (SNBPs), and DNA binding of these proteins [8,24,25]. While our understanding of chronic, low-dose exposures to chemicals is evolving, and the biological mechanisms (germ cell functions, somatic cell functions, reproductive hormone levels/production, hormone receptors, DNA damage, epigenetic modifications, oxidative stress, and inflammation) by which environmental pollution impair spermatogenesis process and sperm functions are being investigated [26,27,28,29,30], it is evident that such exposures are prevalent risk factors for male infertility.

Recent studies suggest that human semen is an overlooked, early, and sensitive marker of both human health and environmental pollution, and the term “semen sentinel” was coined by the group of Montano L. [31,32], who promoted the EcoFoodfertility project, an Italian pilot biomonitoring initiative [33] to assess environmental impact on human health in risk areas.

This work joins the EcoFoodfertility project, and it is the extension of the previous results published elsewhere [22,34,35]. The volatilome of different human body fluid samples (blood, urine, and semen), collected from barely adult boys living in two contaminated areas in Italy, have been analyzed by two sensing technologies (GC/MS and gas sensor array) as novel tools of human biomonitoring to investigate health risk associated with contaminated sites. The two residential areas considered in this study are contaminated sites in Italy known at the European level, i.e., *Land of Fires* (LF) in Campania region (Italy), whose nickname is due to thousands of illegal wastes burned to eliminate traces, and *Valley of Sacco River* (VSR) in Lazio region (Italy), where toxic waste dumps of industrial origin have been poured into the river and used in agriculture and livestock breeding for decades, causing an unprecedented environmental and social disaster.

In this work, we performed an advanced data analysis step where volatilome profiles of blood, urine, and semen samples collected from the above-mentioned pilot study’s male population and analyzed by Headspace–Solid-Phase Microextraction–Gas Chromatography/Mass Spectrometry (HS-SPME-GC/MS) were supported by seminogram analysis carried out on semen samples. Multiple regression models were applied to the data to evaluate the ability of the VOC patterns to predict some seminogram parameters related to sperm count, sperm motility, and sperm morphology.

## 2. Materials and Methods

### 2.1. Subjects

In this study, conducted within the EcoFoodfertility project, a small male population of students (n. 50 subjects in total) of about 18 years old residing in *Land of Fires* (LF) (n. 35 subjects) and *Valley of Sacco River* (VSR) (n. 15) were recruited as volunteers, and their biospecimens were collected (human semen, blood, and urine) after obtaining their informed consent. This study was carried out in accordance with the Code of Ethics of the World Medical Association [36] upon approval of the Ethical Committee of the Local Health Authority Campania Sud-Salerno (protocol number 43_r.p.s.o., 30 June 2015). To exclude special conditions of occupational exposure and consider only general conditions of environmental exposure of the population, only student boys were enrolled. Exclusion factors from this study were the habit of smoking or alcohol consumption, chronic diseases (diabetes or other systemic diseases), malfunctions of the reproductive system (varicocele, prostatitis, etc.), and a high body mass index (BMI > 33 kg/m^2^). Recruitment was carried out at the “San Francesco d’Assisi” Hospital (Oliveto Citra, Salerno, Italy) and the Italian Association of Blood Volunteers (AVIS, Frosinone office, Frosinone, Italy) in 2018.

### 2.2. Sample Collection

Human semen was collected via morning masturbation after 3–4 days of sexual abstinence in sterile containers. Morning fasting blood samples were collected via venipuncture into sodium citrate tubes and shaken gently. The first morning urine samples, also called 8 h samples, were collected after emptying the bladder before going to sleep; each subject, therefore, provided such a urine sample in a sterile 50 mL PVC container. In a subsequent aliquoting phase, aliquots of the different biofluids (250 μL of ejaculated sperm, 1 mL of whole blood, and 1 mL of urine) were taken from standard collection containers and transferred into 5 mL headspace vials (code 27319-U, Shimadzu™, Kyoto, Japan) capped with a screw cap assembled with a hole with a PTFE/silicone septum. The vials were immediately frozen and stored at −80 °C and were sent in batches to the Gas Sensor Lab of the CNR-IMM in Lecce (Italy) for joint analysis of VOCs using gas sensors and Headspace–Solid Phase Microextraction–Gas Chromatography/Mass Spectrometry (HS-SPME-GC/MS).

### 2.3. VOC Analysis

Following a standardized experimental protocol for the treatment of human semen, blood, and urine samples, VOCs were extracted from the headspace of the vials containing the different biological fluids. The frozen samples were thawed at room temperature, and then the vials were immersed in a water bath on a hot plate with a magnetic stirrer at 60 °C overnight. After this incubation, a Carboxen^®^/Polydimethylsiloxane (CAR/PDMS) fiber (57318, Supelco, Bellefonte, PA, USA) was exposed to each sample headspace for 15 min. After the SPME step, the GC/MS analysis of extracted volatiles was performed using GC (6890N series Agilent Technologies, Santa Clara, CA, USA) coupled to MS (5973 series Agilent Technologies, USA) equipped with a ZB-624 capillary column (Phenomenex, Torrance, CA, USA); the SPME fiber was injected into the GC injector and was kept at 250 °C to allow thermal desorption of VOCs. A flow rate of 1 mL/min for the helium carrier gas was used with the following GC temperature program: initial 34 °C held for 2 min and then ramped at 3 °C/min to 110 °C; after that, 5 °C/min to 220 °C held for 2 min. The MS analyses were carried out in full-scan mode with a scan range of 30–500 amu at 3.2 scans/s. The VOCs were searched by non-target analysis, identified by comparing mass spectra with those of the data system library (NIST14, P > 60%), and quantified by the internal standard (I.S.: 1,4-Dichlorobenzene-D4, Sigma Aldrich, St. Louis, MO, USA) method. Details of the recruitment of subjects, sample collection, and VOC analysis by HS-SPME-GC/MS on the different biospecimens have been just published [34,35].

### 2.4. Semen Analysis

Seminogram analysis was carried out on human semen samples collected in sterile containers following the last guidelines and procedures for the examination and processing of human semen given by World Health Organization criteria [37].

A semen analysis or sperm test, also known as a seminogram (or spermogram), is a non-invasive test whose purpose is to evaluate certain characteristics of a male’s semen and the sperm contained therein, allowing for an initial, overall assessment of a man’s fertility based on his semen quality. Thanks to it, the specialist can assess various macroscopic and microscopic parameters. For this study, we used the microscopic data of sperm concentration, motility (percentage of total and progressive motility and immotiles), morphological abnormalities (percentage of total, head, neck, and tail abnormalities), and round cell concentration. The most modern method of semen analysis is the computer-assisted method—the SCA system (Sperm Class Analyzer), which provides fast, accurate, modern, and objective analysis of the key parameters; a phase contrast microscope (Nikon Eclipse TE 300, Tokyo, Japan) was used. The SCA system meets the World Health Organization’s criteria (WHO21) [37,38,39]. WHO21 has established reference values to determine what the normal results should be for a semen analysis report.

According to WHO21 criteria, sperm concentration is the number of sperm per milliliter, and it is expected to be 16 million or more per milliliter.

Sperm are motile cells, and how they move is important. Progressive motility is the spermatozoa’s ability to progress in their advance and, therefore, traverse the fallopian tubes and reach the egg; the spermatozoa must show a progressive motility of 30% or more. Total motility refers to the percentage of sperm making any sort of movement, including non-progressive movements that occur when spermatozoa do not make a forward progression or swim in very tight circles. At least 42% of the sperm should be motile or moving; below 42% is considered low sperm motility. Immotile spermatozoa are unable to move in any way.

Sperm morphology, i.e., the size and shape of sperm, is another factor that is examined as part of a semen analysis; it is reported as the percentage of sperm that appear normal when semen is viewed under a microscope. Normal sperm have an oval head with a long tail. Abnormal sperm have head, neck, or tail defects, such as a large or misshapen head, a bent or irregular neck, or a crooked or double tail. These defects might affect the ability of the sperm to reach and penetrate an egg. However, having a large percentage of misshapen sperm is not uncommon. Typically, only around 4% to 14% of the sperm in a semen sample are normal, meaning that the vast majority do not look perfect under a microscope. The seminogram must show normal sperm forms greater than 4%, which means that the percentage of morphological anomalies must not exceed 96%.

Round cells in seminal fluid are defined as either white blood cells (leucocytes) or immature germ cells (precursors of sperm cells). The presence of leukocytes indicates the presence of an infection, which could lead to alterations in other parameters. Immature germ cells are normally found in the testicles, but sometimes they can be released into the semen. They can be harmless, but they can also indicate problems with sperm maturation or production. They can also affect sperm concentration and morphology [40]. The World Health Organization (WHO21) recommends estimating the concentration of round cells in semen analysis as part of the basic parameters. The normal values are less than 5 million round cells per milliliter of semen and less than 1 million leucocytes per milliliter of semen.

### 2.5. Statistical Data Analysis

Data obtained by both semen and VOC analysis are summarized with descriptive statistics. In an advanced data analysis step, spermiogram parameters and VOCs found in the considered body fluids were processed using statistical regression analysis techniques. The 9 spermiogram parameters (i.e., spermatozoa concentration in 10^6^ cells/mL, progressive motility/total motility/immotiles in percentage, total/head/neck/tail morphological anomalies in percentage, and the round cell concentration in 10^6^ cells/mL) determined by semen analysis were thus used as reference data in multivariate regression modeling, building a predictive model from the set of independent or predictor variables (here, the patterns of the VOC are expressed in ng/mL) to the set of continuous dependent variables (here, the spermiogram parameters). In particular, multiple linear regression (MLR) was used as a multiple regression technique to estimate the relationship between two or more independent variables and one dependent variable. We used it to determine (a) how strong the relationship is between the predictor variables (VOC concentration) and each dependent variable (spermiogram parameters) and (b) the predicted value of the dependent variable at certain values of the independent predictor variables.

All the statistical data analyses were carried out by Statistics for Data Analysis (SPSS v. 29.0.1) and OriginPro 8.6.

## 3. Results

### 3.1. Descriptive Statistics of Male Population Seminograms

**Figure 1:**
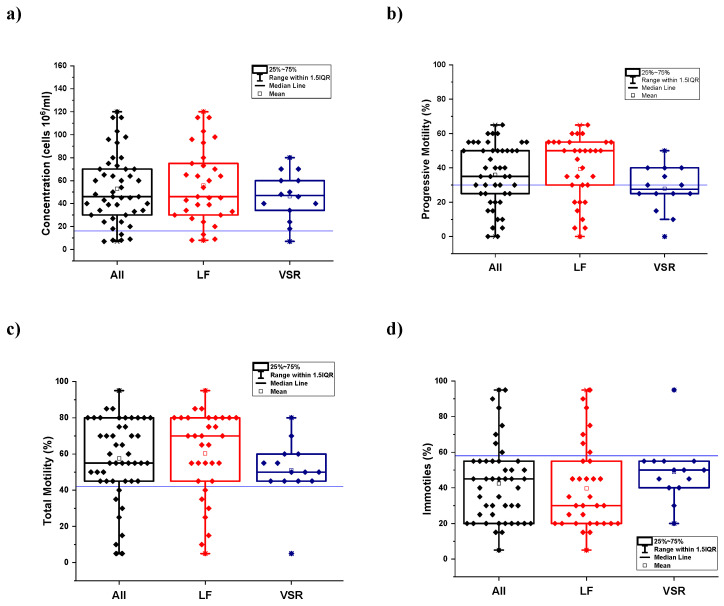
Box and whisker plots displaying the dispersion of the seminogram parameters related to sperm count and motility: (**a**) sperm cell concentration; (**b**) progressive motility; (**c**) total motility; (**d**) immotile cells in the subgroups of the population living in *Land of Fires* (LF) and in *Valley of Sacco river* (VSR) and the total population (all = LF + VSR).

The data obtained by semen analysis carried out on the sample population living in *Land of Fires* (LF) and *Valley of Sacco River* (VSR) were first explored by descriptive statistics. Box and whisker plots have been used for all the seminogram parameters as a graphical representation to describe data distribution and skewness in both subgroups (LF, VSR) and whole (all = LF + VSR) sample population through simple dispersion and position indices. Box and whisker plots displaying the dispersion of sperm count and motility are shown in Figure 1, whereas those displaying sperm anomalies and round cell concentrations are shown in Figure S1. The box indicating the interquartile range (IQR, middle 50% of our population) within the 25–75% quartiles, the central tendency (median and mean), the whiskers extending to 1.5 IQR, and the outliers have been computed for each group of cases. In the plots for each seminogram parameter, the reference lines (blue lines) indicate the threshold value above or below which the parameter is in the normal range.

In all box and whisker plots, it can be observed that although most of the data fall within normal ranges, there is a non-negligible part of data outside these ranges, which constitutes an alert factor.

Some cases (subjects) show a sperm count below the threshold value of 16 million cells per milliliter (Figure 1a). Regarding motility parameters, many observations are below the reference line; in the box and whisker plot of the sperm progressive motility that must be 30% or more, the reference line crosses the box plot, and a significant part of cases are below this threshold value (Figure 1b). Regarding sperm total motility, which also includes non-progressive movements and the complementary immotile spermatozoa, part of the sample population shows a sperm total motility below the cutoff value of 42% (Figure 1c) and, correspondingly, a percentage of immotile cells above 58% (Figure 1d).

Sperm morphological anomalies in the sample population have a high percentage but for the majority of cases, they are below the tolerable value of 96% (total sperm anomalies), and in a smaller but not insignificant part of cases they exceed this value. However, to evaluate semen sample quality, some laboratories still use Kruger’s strict criteria (WHO99) [41], which is far stricter than the ones published by WHO10 [42] and WHO21 [37]. According to Kruger’s strict criteria, teratozoospermia is present when more than 86% of spermatozoa have an abnormal shape. In other words, the borderline to consider whether a man has teratozoospermia is set at a minimum of 14% of spermatozoa with normal forms instead of 4%, which is the percent currently established by WHO21 [39,43]. It is significant that almost all our samples exceed the limit set by the Kruger criterion (Figure S1a).

It can be also observed that among the different types of morphological sperm anomalies, those present in the highest percentage are those of the head (Figure S1b), followed by those of the neck (Figure S1c) and tail (Figure S1d). Sperm pathologies primarily affecting the head–neck and secondary tail impair sperm function; on a cellular basis, the hidden causes behind a head shape change play a significant role in fertilization.

Furthermore, a significant portion of the sample population was found to have a concentration of round cells equal to or greater than the limit of 5 million per milliliter set by the WHO21 criteria (Figure S1e). Although the size of the sample population is small, what emerges from this sperm characterization of boys residing in the two contaminated areas considered in this study is that there is a fraction of the sample population with poor sperm quality and not within the limits recommended by the World Health Organization. What is of concern in these data is the young age of the recruited subjects, as all boys are just over 18 at the peak of their reproductive capacity; one would not expect the first signs of deterioration of sperm parameters from them. In line with this experimental evidence, other studies on the same contaminated areas have recorded molecular alterations in the sperm of young people and metal contamination [25,44].

### 3.2. Data Analysis by Regression Methods

In this study, the joint use of GC/MS and seminograms gave the opportunity to relate the volatilome fingerprinting of the considered biosamples in terms of the most significant VOCs identified in the headspace of the considered biosamples (blood, urine, and semen) to the corresponding seminogram parameters. Specifically, 42 statically relevant VOCs identified and quantified by the HS-SPME-GC/MS analysis were combined with seminogram parameters. The rationale for this study is that the VOC profile of blood (B), urine (U), and sperm (HS), which essentially represents the health status of a subject based on his internal and external exposome, can be indicative not only of pathological conditions (applicable in diagnostics) but also of multiple environmental exposures that can contribute to compromising the general quality of sperm. Human exposome and human volatilome are strictly interconnected, and their hidden relationship could be exploited both to discriminate populations living in areas at high vs. low pollution impacts as the index of risk for health and to indirectly estimate clinical or physiological parameters. Looking at this last potential use, we pushed ourselves to evaluate the feasibility of the predictive potential of specific VOC patterns of human biospecimens towards variables of interest, such as spermiogram parameters, using multiple linear regression (MLR) analysis.

According to the VOC distribution in human semen, blood, and urine, some VOCs are common to all three biofluids (HS, B, and U) and others to two of them, whereas others are only present in one biofluid; thus, the forty-two statistically relevant VOCs selected by HS-SPME-GC/MS give rise to twenty-two VOC variables for HS, twenty-nine for B, and twenty-six for U. Multiple linear regression (MLR) analysis was hence applied to all nine spermiogram parameters used as dependent variables. Initially, all VOC variables were used as predictor variables in the MLR analysis, and different models were built, depending on which biofluids (1 or 2 or all 3) were included in the data analysis, i.e., human semen and blood and urine (HS + B + U), human semen and blood (HS + B), human semen and urine (HS + U), human semen only (HS), blood only (B), and urine only (U).

In model building, all effects (forward stepwise, backward stepwise, forward entry, backward removal, and best subsets) were tested. In each model and for each spermiogram parameter, a Pareto chart of the t-values for the regression coefficients was used as a guide for selecting the optimal predictors. The Pareto chart shows the parameter estimates (or t-values) sorted by their absolute size. On the Pareto chart, bars that cross the reference line (corresponding to the current criterion of statistical significance *p* < 0.05) are statistically significant; by working on retaining only the most significant predictive variables, a more performing regression model was refined based on the patterns of those VOCs that contribute more to the predictive model. We list all the VOCs that were found to be significant as predictive variables in the statistical regression analysis (VOC predictors) in a supplementary table (Table S1) with the values relating to the level of concentration and distribution in the sample population.

It is worth noticing that in this way, starting from volatilome characterization of human biofluids, such as blood, urine, and semen, novel VOC-based models for the estimation of the spermiogram parameters were developed.

Figure 2a, Figure 3a, Figure 4a, Figure 5a and Figures S2a–S6a show the overall fit of the MLR model described by the test of the SS whole model vs. SS residual for all the nine spermiogram parameters estimated by the VOC-based model with a significant *p*-value (*p* < 0.05).

Each table reports the results for a predicted spermiogram parameter comparing the different groups of data (HS + B + U; HS + B; HS + U; B + U; HS; B; U); specifically, the multiple R, R^2^, adjusted R^2^, and overall F-test results were displ ayed for each group of data. The multiple R is the coefficient of multiple correlations, which is the positive square root of R-square (the coefficient of multiple determination); the R^2^ value is an indicator of how well the model fits the data (e.g., an R^2^ close to 1.0 indicates that we have accounted for almost all of the variability with the variables specified in the model); and the adjusted R^2^ is interpreted similarly to the R^2^ value, except the adjusted R^2^ takes into consideration the number of degrees of freedom. The overall F-test determines whether the relationship between the dependent variable and the set of independent variables is statistically significant; if the *p*-value for the overall F-test is less than the significance level, you can conclude that the R^2^ value is significantly different from zero.

**Figure 2:**
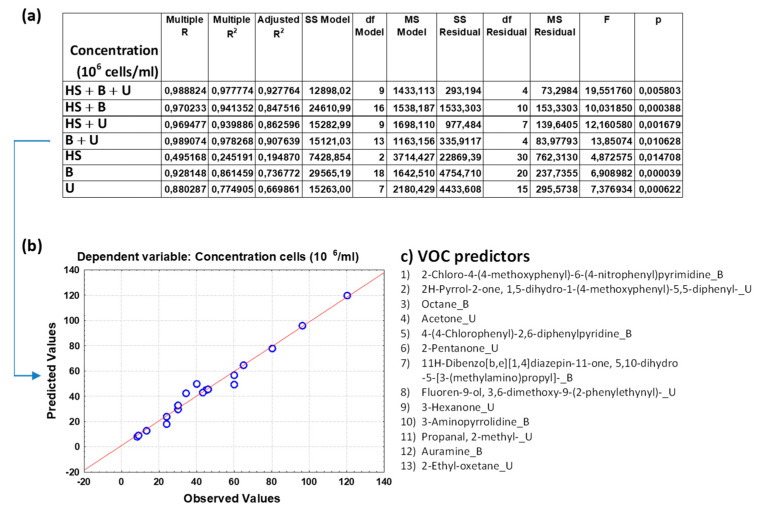
(**a**) Overall fit of the MLR model described by the test of the SS whole model vs. SS residual for sperm concentration (in 10^6^/mL); (**b**) observed vs. predicted values for sperm concentration as a result of the MLR analysis for the data group B + U based on selected VOC predictors; (**c**) pattern of VOCs used as predictor variables in the MLR model as selected by a Pareto chart; the order in the list reflects the greatest predictive contribution to the model (VOC name_X; X = B, blood, U, urine, or HS, human semen). Notation for numeric values: comma “,” is the decimal separator (SI).

**Figure 3:**
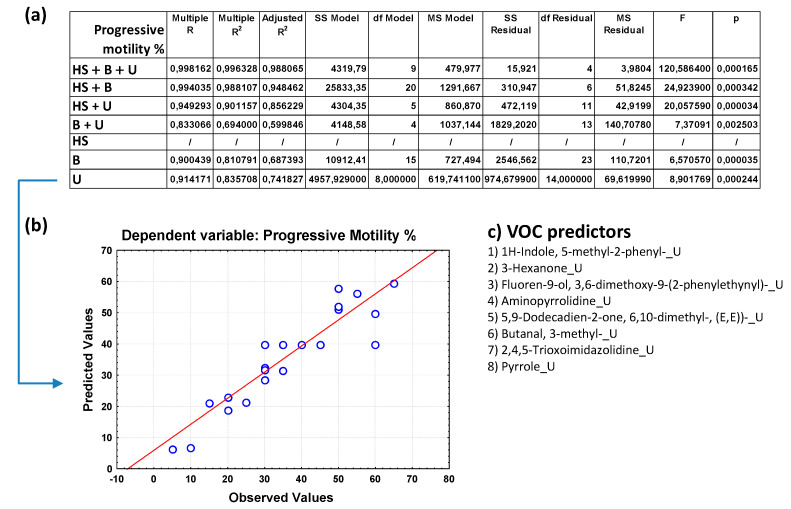
(**a**) Overall fit of the MLR model described by the test of the SS whole model vs. SS residual for sperm progressive motility (in percentage); (**b**) observed vs. predicted values for sperm progressive motility as a result of the MLR analysis for the data group U based on selected VOC predictors; (**c**) pattern of VOCs used as predictor variables in the MLR model as selected by a Pareto chart; the order in the list reflects the greatest predictive contribution to the model (VOC name_X; X = B, blood, U, urine, or HS, human semen). Notation for numeric values: comma “,” is the decimal separator (SI).

**Figure 4:**
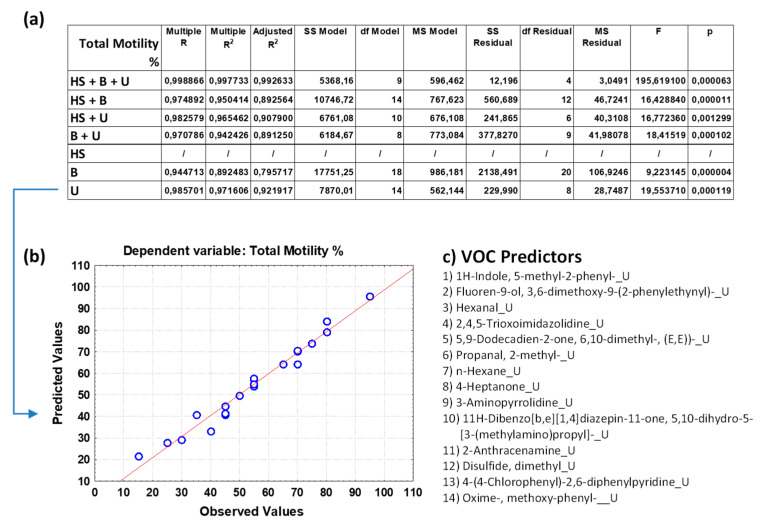
(**a**) Overall fit of the MLR model described by the test of the SS whole model vs. SS residual for sperm total motility (in percentage); (**b**) observed vs. predicted values for sperm total motility as a result of the MLR analysis for the data group U based on selected VOC predictors; (**c**) pattern of VOCs used as predictor variables in the MLR model as selected by a Pareto chart; the order in the list reflects the greatest predictive contribution to the model (VOC name_X; X = B, blood, U, urine, or HS, human semen). Notation for numeric values: comma “,” is the decimal separator (SI).

**Figure 5:**
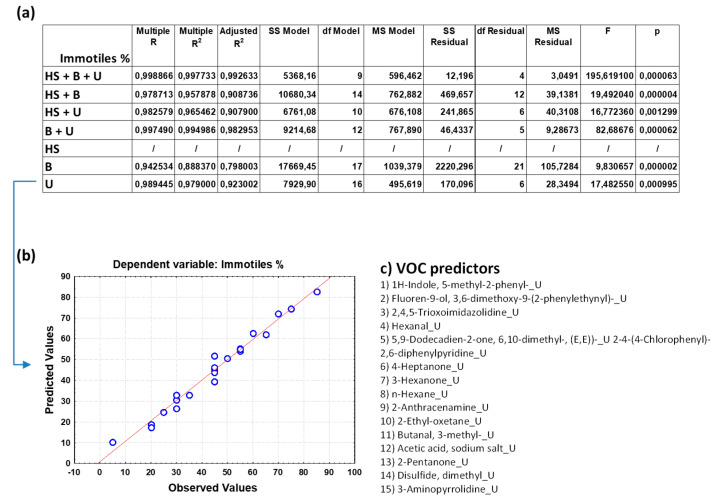
(**a**) Overall fit of the MLR model described by the test of the SS whole model vs. SS residual for sperm immotiles (in percentage); (**b**) observed vs. predicted values for sperm immotiles as a result of the MLR analysis for the data group U based on selected VOC predictors; (**c**) pattern of VOCs used as predictor variables in the MLR model as selected by a Pareto chart; the order in the list reflects the greatest predictive contribution to the model (VOC name_X; X = B, blood, U, urine, or HS, human semen). Notation for numeric values: comma “,” is the decimal separator (SI).

The comparison of the overall fit of the multiple regression model between the different data groups (Figure 2a, Figure 3a, Figure 4a, Figure 5a and Figures S2a–S6a) allows us to highlight some surprising results and make some considerations on the great potential of the analysis of VOCs in human biofluids. Although the MLR model that considers the trio of biofluids (HS + B + U) generally has a better fit for most sperm parameters than regression models that consider only a duo of biofluids or those that consider only one, the predictive power of the latter remains very high. Unexpectedly it can be observed that the HS data group is not efficient in estimating sperm concentration, whereas when combined with B and/or U data, the predictive power also increases with the contribution of the VOCs found in HS. Attention is focused on the binary model that considers blood and urine (B + U) data and on the single models that consider blood (B) data and urine (U) data. Although these models exclude the VOC variables of the human semen from the multiple regression analysis, they unexpectedly allow us to estimate the value of the different sperm parameters with a good fitting. In particular, the B + U data group can be considered sufficient to evaluate sperm concentration with a high multiple R coefficient for most of the spermiogram parameters, as evident from good linear data distribution in the graph of observed vs. predicted values. However, some sperm parameters are better or equally estimated by the U data group compared to the B + U data group.

To give greater emphasis to the application potential of these results, we decided to show the predicted vs. observed graphs resulting from the MLR analysis based on the U data subgroup for the following six spermiogram parameters (total motility, progressive motility, immotile cells, head and neck anomalies, and round cells, respectively, in Figure 3b, Figure 4b, Figure 5b and Figures S3b, S4b and S6b) and those obtained with the B + U data subgroup for the remaining three spermiogram parameters (cells concentration, morphology anomalies, and tail anomalies, respectively, in Figure 2b, Figures S2b and S5b). Contextually, for the mentioned MLR analysis based on the specific U and B + U data subgroups for the different sperm parameters, Figure 2c, Figure 3c, Figure 4c, Figure 5c and Figures S2c–S6c report the corresponding pattern of VOCs used as independent predictive variables in the regression model as selected by a Pareto chart; the order in the list reflects the greatest predictive contribution to the model (VOC name_X; X = B, blood, U, urine, or HS, human semen). The detailed MLR results related to the HS + B + U dataset are shown in Figures S7–S15; the predictive ability of the different spermiogram parameters is constructed with the contribution of VOCs found in the three biofluids.

## 4. Discussion

The role of the VOCs found in the biofluids considered in the work and used as predictive variables in the regression model was explored in depth on the basis of some databases that collect the VOC compounds present in the human volatilome: the Human Metabolomic Database (HMDB version 5.0) [45,46]; the CompTox Chemicals Dashboard v2.3.0 [47,48] by the United States Environmental Protection Agency (EPA); the Comparative Toxicogenomics Database [49]; and the PubChem Compound database [50].

It is worth arguing that assessing chemicals for their potential to cause male reproductive toxicity involves the evaluation of evidence obtained from experimental, epidemiological, and mechanistic studies. For male reproductive toxicants, eight key characteristics were identified based on a survey of established mechanisms and include alterations in (1) germ cell functions, (2) somatic cell functions, (3) reproductive hormone levels/production, (4) hormone receptors, (5) DNA damage, (6) epigenetic modifications, (7) oxidative stress, and (8) inflammation. All these pathways contribute to the pathogenesis of impaired male fertility potential [27,28,29,30,51,52].

Regarding sperm concentration, we can comment on the contribution to parameter estimation given by blood and urinary VOC predictors (Figure 2c). First, a main contribution is given by pyridine derivates and pyrimidines. Pyridine is a compound considered carcinogenic; it is often used as a denaturant for antifreeze mixtures, ethyl alcohol, and fungicides, as well as a dyeing aid for textiles. It is a harmful substance if inhaled, ingested, or absorbed through the skin, and it also reduces male fertility. Pyrimidine is a heterocyclic aromatic organic compound similar to benzene and pyridine; pyrimidines serve essential functions in human metabolism. Pyrrole itself is not naturally occurring, but many of its derivatives are found in a variety of cofactors and natural products; pyrrole and pyrrole-based compounds are largely used as flavoring and pharmaceutical ingredients, as well as ingredients for pesticides and insecticides. Octane is a potentially toxic compound hydrocarbon. Ketones, such as acetone, 2-pentanone, and 3-hexanone, mainly come from diet and have been found to be associated with several diseases. Fluorenes are potentially toxic compounds containing a fluorene moiety, which consists of two benzene rings connected through either a cyclopentane, cyclopentene, or cyclopenta-1,3-diene. Auramine is not a naturally occurring metabolite and is only found in those individuals exposed to this compound or its derivatives; it is a known human carcinogen [53]. Propanal, 2-methyl- is an aldehyde with many toxicologies (chronic, subchronic, developmental, genotoxicity).

Looking at the urinary VOC predictors of sperm progressive motility, we can find some of the mentioned VOC predictors (3-hexanone, pyrrole-based, fluorenes) and other exogenous compounds, such as 1H-Indole,5-methyl-2-phenyl, 5,9-Dodecadien-2-one, 6,10-dimethyl-, (E,E))-, 2,4,5-trioxoimidazolidine, butanal, and 3-methyl (Figure 3c). The latter is found in low concentrations in many types of food, and commercially, it is used as a reagent to produce pharmaceuticals, perfumes, and pesticides.

Regarding total sperm motility (Figure 4c), it is interesting to note among the urinary VOC predictors, apart from some predictive VOCs common to the previous sperm parameters, the presence of hexanal, which is an alkyl aldehyde found in human biofluids with genotoxic and cytotoxic properties, as well as of n-hexane, which is a gasoline tasting compound found in food; it is potentially toxic, causing degeneration of the peripheral nervous system, and exposure to it may also damage the lungs and reproductive system. 4-Heptanone, belonging to ketones, is considered to be an oxygenated hydrocarbon lipid molecule that is practically insoluble in water. 2-Aminoanthracene, a genotoxic chemical belonging to anthracenes (organic compounds containing a system of three linearly fused benzene rings), was also registered; it is not a naturally occurring metabolite and is only found in individuals exposed to this compound or its derivatives. Dimethyl disulfide (DMDS) is widespread in nature, as it is emitted by bacteria, fungi, plants, and animals, and it is used as a food additive, industrial sulfiding agent, and effective soil fumigant in agriculture.

As expected, in the model for immotile sperm cell estimation based on urinary VOCs, we found many of the VOC predictors of the previous sperm motility parameters (Figure 5c).

These results suggest hidden mechanisms in which predictive VOCs are representative of sperm cell concentration and play a role in determining their motility characteristics. Since many of the VOCs found in biofluids belong to human exposome, their influence on semen quality is indicative of excessive exposure to multiple environmental stressors and could be significant in expanding the list of chemicals of emerging concern and biomarkers with health effects in human biomonitoring.

The role of VOCs, selected from the volatilome of the considered biofluids in the deterioration of sperm quality, also extends to morphological abnormalities and round cells. We also observe that curiously, the regressive model based on the blood dataset (B) is not suitable for estimating the total morphological anomalies of the sperm, those of the head and those of the neck, or round cells, while it fairly estimates the anomalies of the tail. For this reason, it was decided to indicate the results deriving from the model based on the B + U dataset for morphological anomalies (Figure S2) and tail anomalies (Figure S5) and the one based on the U dataset for head (Figure S3) and neck anomalies (Figure S4), as well as round cells (Figure S6). It should also be noted that the regression model based on the human semen dataset (HS) with the related panel of semen VOC predictors performs less well than the models based on the B + U and U datasets.

Looking at total morphological anomalies, we found compounds as VOC predictors belonging to pyrroles, pyridine and fluorenes, hexanal, 2-anthracenamine, and other chemicals, such as propane, 2-(ethenyloxy)-, oxime-,methoxy-phenyl-, 2-ethyl-oxetane, and 1-(6-Methyl-benzothiazol-2-yl)-3-(4-methyl-benzoyl)-thiourea (Figure S2c).

Many compounds found in the previous model are also VOC predictors of head morphological anomalies; as novel predictors, we mentioned the main role of 11H-Dibenzo[b,e][1,4]diazepin-11-one, 5,10-dihydro-5-[3-(methylamino)propyl], and secondary butanal (Figure S3c). Butanal is an aldehyde that exists in all living organisms, ranging from bacteria to humans, and outside of the human body, it is found in several different foods.

Regarding VOC predictors of neck morphological anomalies, we can observe, as not yet mentioned, the contribution of 2-Butanone (Figure S4c). It has been detected in several different foods, and it is also a secondary metabolite; with regard to humans, has been found to be associated with several diseases, such as Crohn’s disease, pervasive developmental disorder not otherwise specified, asthma, and ulcerative colitis; butanone has also been linked to the inborn metabolic disorder celiac disease.

Regarding VOC predictors of tail morphological anomalies, we can observe, as not yet mentioned, the contribution of Benzaldehyde,2-nitro-,diaminomethylidenhydrazone and alpha-Pinene (Figure S5c). The latter is an organic compound of the terpene class and is found in the oils of many species of many coniferous trees; it is also found in cannabis plants and in finished, dried cannabis flower preparation, commonly known as marijuana.

Finally, looking at the VOC predictors of round cells, many of the compounds predictive of the other spermiogram parameters are present (Figure S6c).

It is also worth discussing the results of the regression models that consider the VOC variables identified in the human semen, in particular the model based on the HS + B + U data, to evaluate which sperm VOCs were selected by the model as predictive variables. Looking at the results related to the HS + B + U dataset and shown in Figures S7–S15, we can observe that some VOCs of HS with toxic properties played a role in the prediction model, together with the VOCs of blood and urine. Among the VOC predictors for the HS + B + U model, we highlight the following VOCs of HS (label VOCname_HS): (a) 1-anthracenamine; propane, 2-(ethenyloxy); 3-aminopyrrolidine; and 2-ethyl-oxetane (for sperm cells concentrations, Figure S7); (b) acetic acid; sodium salt; hexanal; butanal; and 2-methyl-; 1-anthracenamine (for sperm progressive motility, Figure S8); (c) 1-anthracenamine; n-hexane; butanal; and 2-methyl- (for sperm total motility, Figure S9); (d) 1-anthracenamine; butanal and 2-methyl- (for sperm immotiles, Figure S10); (e) n-hexane (for sperm morphology anomalies, Figure S11); (f) auramine and hexanal (for sperm head anomalies, Figure S12); (g) acetic acid; sodium salt; butanal; and 2-methyl- (for sperm neck anomalies, Figure S13); (h) butanal; pentanal; acetic acid; sodium salt; and oxime,methoxy-phenyl- (for sperm tail anomalies, Figure S14); and (i) acetone; n-hexane; and pentanal (for round cell concentration, Figure S15).

To have an overview of all VOC variables that emerged as having a predictive role in panels based on the analyses carried out, Table S2 summarizes all the VOC predictors in the different regression models (based on datasets HS + B + U; HS + B; HS + U; B + U; HS; B; U). Since a compound was found in more than a biofluid, for each VOC predictor, the origin biofluid jointed to the related dataset of the regression models is reported, in which it was a predictive variable. Moreover, Table S2 shows the CAS number of the VOCs and the relevant chemical dashboards in the freely available electronic database used in this work, which offer rich information on the VOCs’ properties and their harmful effects recorded so far.

## 5. Conclusions

This pilot biomonitoring study addresses the issue of health risk assessment in populations in contaminated areas. In this work, we contributed to demonstrating that the human volatilome contains fundamental and hidden information about the organism’s reactions to environmental exposures, particularly toxic chemical exposures that negatively impact health.

The results of this work propose an original approach to exploit the correlations between the human volatilome and other measurable health effects, such as spermiogram parameters, which are indicative of sperm quality. The idea is to use the general regression model based on optimized panels of VOCs as independent variables in different biofluids (sperm, blood, urine) as an indirect measure of the spermiogram parameters. Such an innovative approach can be extended to other areas of the human exposome for the estimation of other clinical parameters as well as known or company biomarkers that describe the health status of a subject. In this way, the appropriate use of the data analysis technique contributes to validating the panels of VOCs, selected from the biofluids’ volatilome, as new candidates for biomarkers of exposure and/or disease risk.

Furthermore, it should be underlined that it is certainly encouraging to observe the good performance of MLR models that do not use sperm data to estimate spermiogram parameters. The implications of this result are very interesting, as they have strong implications for preventive reproductive medicine together with a strong social impact. In fact, a simple urine or blood sample may be sufficient to obtain an indirect assessment of sperm quality, thus also overcoming the psychological stress and anxiety associated with sperm analysis in young men. VOC analysis of blood, urine, and (optionally) semen can hence be a new methodology for the analysis of semen quality. Sperm analysis is indeed usually prescribed to men only when seeking paternity within a couple’s relationship. Postponing this test and an andrological visit to a more adult age effectively prevents the screening of boys who already show signs of sperm deterioration at a young age and on whom it is appropriate to carry out more complete medical investigations.

It is beyond the scope of this study to hypothesize possible biological pathways of toxicity involving the VOCs identified as predictive, for which we refer to the database platforms that collect all the data available for a deeper discussion. In this study, we limit reporting on the association between VOCs found in body fluids and the detrimental impacts on sperm parameters. It is difficult to explain the precise molecular mechanisms of those significant VOCs in eliciting adverse health effects in populations, as they have not always been fully elucidated. Anyway, a survey of some key characteristics has been identified, involving (1) alterations in germ cell development, functions, or death; (2) alteration in somatic cell development, functions, or death; (3) alterations in production and levels of reproductive hormones; (4) alterations of hormone receptor levels/functions; (5) genotoxic DNA damage, chromosome fragmentation, altered sperm cell chromosome numbers; (6) induction of epigenetic alterations; (7) induction of oxidative stress; and (8) induction of inflammation [27,28,29,30,51,52].

However, it is necessary to point out that although the predictive capacity of the VOCs identified in biofluids regarding sperm concentration, motility, shape anomalies, and round cells leads to the hypothesis of more potent biochemical interactions within body fluids, which ultimately affect sperm parameters, it is not possible to draw causal conclusions now, but it is legitimate to consider the presence of predictive VOCs as an alarm signal for sperm quality.

This work also lays the foundations for exposome studies that include innovative volatilomics studies on human biofluids; the latter can offer a tool for investigating possible health risks and for measuring parameters of clinical interest that are subtly affected by chemical exposure factors. The point of strong debate in the scientific field relating to how to demonstrate the impact of chemical exposure resulting from external factors would, hence, make use of a new analysis method for human biomonitoring based on volatilomics, which would validate the approach both as an explorer of new biomarkers and as a measurement method of its effects on health through general regression models combined with other biomarkers or measurable parameters. The idea is that the human volatilome with all VOCs, both endogenous and exogenous, contains information on actual health, and this concept can, therefore, also be extended to volatilomics applied to early diagnostics.

## Data Availability

The data presented in this study are available on request from the corresponding author due to privacy and ethical reasons.

## Supplementary Materials

The following supporting information can be downloaded at https://www.mdpi.com/article/10.3390/toxics12080543/s1, Figure S1: Box and whisker plot displaying the dispersion of the seminogram parameters related to sperm morphology and round cells. Figure S2: Results of the MLR model for sperm morphology anomalies for the data group U; Figure S3: Results of the MLR model for sperm head anomalies for the data group U; Figure S4: Results of the MLR model for sperm neck anomalies for the data group U; Figure S5: Results of the MLR model for sperm head anomalies for the data group U; Figure S6: Results of the MLR model for round cells for the data group U; Figure S7: Results of the MLR model for sperm concentration for the data group HS + B + U; Figure S8: Results of the MLR model for sperm progressive motility for the data group HS + B + U; Figure S9: Results of the MLR model for sperm total motility for the data group HS + B + U; Figure S10: Results of the MLR model for sperm immotiles for the data group HS + B + U; Figure S11: Results of the MLR model for morphology anomalies for the data group HS + B + U; Figure S12: Results of the MLR model for sperm head anomalies for the data group HS + B + U; Figure S13: Results of the MLR model for sperm neck anomalies for the data group HS + B + U; Figure S14: Results of the MLR model for sperm tail anomalies for the data group HS + B + U; Figure S15: Results of the MLR model for round cells for the data group HS + B + U; Table S1: List of VOC predictors. Table S2: Characteristics of VOC predictors.

## Abbreviations

VSR	Valley Of Sacco River
LF	Land of Fires
HS	Headspace
SPME	Solid-Phase Microextraction
GC	Gas Chromatography
MS	Mass Spectrometry
VOCs	Volatile Organic Compounds
PAH	Polycyclic Aromatic Hydrocarbons
DNA	Deoxyribon Nucleic Acid
PVC	Polyvinyl Chloride
PTFE	Polytetrafluoroethylene
SCA	Sperm Class Analyzer
WHO	World Health Organization
CAS	Chemical Abstracts Service registry number
MLR	Multiple Linear Regression
IQR	Interquartile Range
