# Supplementary material for: Innovative Approach for Human Semen Quality Assessment Based on Volatilomics
Source: Toxics. 2024 Jul 27;12(8):543. doi: 10.3390/toxics12080543 (PMC11360181; doi:10.3390/toxics12080543)
Supplement: Supplementary file 1 [file toxics-12-00543-s001.zip › toxics-3068851-supplementary.pdf]

Supplementary

Innovative Approach for Human Semen Quality Assessment Based on Volatilomics

Simonetta Capone [1,*], Angiola Forleo [1], Antonio Vincenzo Radogna [1,2], Valentina Longo [1], Giulia My [1], Alessandra Genga [3], Alessandra Ferramosca [2], Giuseppe Grassi [4], Flavio Casino [1], Pietro Siciliano [1], Tiziana Notari [5], Sebastiana Pappalardo [6], Marina Piscopo [7] and Luigi Montano [8,9,10*]

[1] National Research Council, Institute for Microelectronics and Microsystems (CNR-IMM), 73100 Lecce, Italy; angiola.forleo@cnr.it (A.F.); antonio.radogna@unisalento.it (A.V.R.); valentinalongo2110@gmail.com (V.L.); my.giulia@imm.cnr.it (G.M.); flavio.casino@cnr.it (F.C.); pietroaleardo.siciliano@cnr.it (P.S.)
[2] Department of Experimental Medicine, University of Salento, 73100 Lecce, Italy; alessandra.ferramosca@unisalento.it
[3] Department of Biological and Environmental Sciences and Technologies, University of Salento, 73100 Lecce, Italy; alessandra.genga@unisalento.it
[4] Department of Engineering for Innovation, University of Salento, 73100 Lecce, Italy; giuseppe.grassi@unisalento.it
[5] Reproductive Medicine Unit of Check Up Polydiagnostic Center, 84131 Salerno, Italy; tiziananotari7@gmail.com
[6] Reproduction and Fertility Center, 00128 Rome, Italy; pappalardo@riproduzionefertilita.it
[7] Department of Biology, University of Naples Federico II, 80138 Naples, Italy; marina.piscopo@unina.it
[8] Andrology Unit and Service of Lifestyle Medicine in UroAndrology, "S. Francesco d'Assisi" Hospital, Oliveto Citra, 84020 Salerno, Italy
[9] Coordination Unit of the Network for Environmental and Reproductive Health (EcoFoodFertility Project), "S. Francesco d'Assisi" Hospital, Oliveto Citra, 84020 Salerno, Italy
[10] Department of Biology, Tor Vergata University of Rome, 00133 Rome, Italy
* Correspondence: simonetta.capone@cnr.it (S.C.); l.montano@aslsalerno.it (L.M.); Tel.: +39-0832-422507 (S.C.)

Table S1 List of VOC predictors. For each VOC it's reported the concentration in ng/ml in the biofluid where it was found (HS: Human Semen; B: Blood; U: Urine), the mean, standard deviation (SD), median, upper and lower limit, and the 95th percentile in the sample population. Notation for numeric values: comma "," is the decimal separator (SI).

n.	VOC predictor	BIOFLUIDS (HS, B, U)	MEAN (ng/ml)	SD (ng/ml)	MEDIAN (ng/ml)	CI 95%		
						LOWER LIMIT (ng/ml)	UPPER LIMIT (ng/ml)	PERCENTILE 95th (ng/ml)
1	**(3-Methoxy-phenyl)-(6-methyl-4-phenyl-quinazolin-2-yl)-amine**	HS	4,84	19,38	0	0	84,84	77,78
		B	6,62	24,74	0	0	132,11	79,77
2	**1-(6-Methyl-benzothiazol-2-yl)-3-(4-methyl-benzoyl)-thiourea**	HS	7,54	24,69	0	0	100,28	90,59
		B	4,77	20,62	0	0	127,7	29,36
3	**11H-Dibenzo[b,e][1,4] diazepin-11-one, 5,10-dihydro-5-[3-(methylamino) propyl]-**	B	1,53	4,54	0	0	22,22	12,61
		U	0,60	1,79	0	0	7,73	6,84
4	**1-Anthracenamine**	HS	9,98	27,44	0	0	87,88	87,77
5	**1H-Indole, 5-methyl-2-phenyl-**	U	0,24	0,80	0	0	2,84	2,81
6	**2,4,5-Trioxoimidazolidine**	U	1,01	2,27	0	0	6,64	6,49
7	**2-Anthracenamine**	HS	16,54	39,64	0	0	177,16	118,86
		B	3,17	9,12	0	0	33,91	33,26
		U	1,96	4,65	0	0	17,35	15,96
8	**2-Butanone**	U	6,97	12,64	0	0	38,22	37,19
9	**2-Chloro-4-(4-methoxyphenyl)-6-(4-nitrophenyl)pyrimidine**	B	3,69	11,42	0	0	58,57	32,85
10	**2-Ethyl-oxetane**	HS	31,87	102,01	0	0	523,56	324,84
		B	241,25	1368,04	0	0	8767,93	463,77
		U	1,48	2,83	0	0	9,89	9,39

**11**	**2H-Pyrrol-2-one, 1,5-dihydro-1-(4-methoxyphenyl)-5,5-diphenyl-**	B	2,60	9,47	0	0	46,83	35,16
		U	0,58	2,00	0	0	8,57	7,79
**12**	**2-Pentanone**	B	6,48	22,18	0	0	135,45	29,99
		U	33,98	51,12	12,4	0	165,26	163,75
**13**	**3-Aminopyrrolidine**	HS	6,23	20,24	0	0	80,76	72,11
		B	0,63	2,82	0	0	14,02	10,54
		U	1,84	4,73	0	0	19,53	17,67
**14**	**3-Hexanone**	U	1,31	3,06	0	0	10,07	9,83
**15**	**4-(4-Chlorophenyl)-2,6-diphenylpyridine**	HS	10,44	35,22	0	0	142,52	138,54
		B	6,63	21,46	0	0	111,15	66,19
		U	4,30	8,65	0	0	25,17	24,69
**16**	**4-Heptanone**	U	70,11	163,62	23,01	0	773,91	663,82
**17**	**5,9-Dodecadien-2-one, 6,10-dimethyl-, (E,E))-**	U	1,72	5,91	0	0	25,06	22,93
**18**	**Acetic acid, sodium salt**	HS	7,97	27,69	0	0	135,8	90,47
		B	6,74	29,35	0	0	182,61	42,50
		U	6,24	26,35	0	0	126,71	103,19
**19**	**Acetone**	HS	17,39	218,93	128,92	0	1109,2	695,16
		B	3432,27	2898,01	3803,49	0	8767,93	7802,82
		U	115,87	190,99	52,99	0	897,39	769,88
**20**	**alpha-Pinene**	B	2,64	7,30	0	0	34,12	24,03
**21**	**Auramine**	HS	11,94	25,78	0	0	73,08	69,13
		B	0,98	3,63	0	0	17,52	12,56
**22**	**Benzaldehyde, 2-nitro-, diaminomethylidenhydrazone**	B	1,65	4,64	0	0	19,26	15,20
		U	0,59	1,70	0	0	7,14	6,38
**23**	**Butanal**	HS	8,28	27,45	0	0	117,2	100,32
		U	0,84	2,05	0	0	7,97	7,28

**24**	**Butanal, 2-methyl-**	HS	26,32	41,50	0	0	111,12	106,92
**25**	**Butanal, 3-methyl-**	HS	73,72	54,67	74,12	0	173,76	173,34
		B	2,57	7,58	0	0	34,89	22,29
		U	1,67	3,81	0	0	15,1	13,91
**26**	**Butane, 2-methyl-**	HS	21,20	60,18	0	0	246,12	230,33
		B	0,91	4,21	0	0	23,43	12,58
**27**	**Cyclohexane**	B	23,37	43,60	0	0	223,37	124,36
**28**	**Cyclopentane, methyl-**	B	109,80	122,30	77,41	0	418,62	398,52
**29**	**Disulfide, dimethyl**	U	31,74	58,48	11,74	0	268,57	324,86
**30**	**D-Limonene**	HS	80,89	296,67	0	0	1353,56	1163,05
		B	1,13	5,13	0	0	26,89	17,59
**31**	**Fluoren-9-ol, 3,6-dimethoxy-9-(2-phenylethynyl)-**	HS	34,74	49,75	0	0	208,44	138,78
		U	1,30	3,67	0	0	14,76	13,57
**32**	**Heptanal**	B	0,60	2,67	0	0	12,87	10,46
**33**	**Heptane**	B	10,19	27,39	0	0	150,9	71,29
**34**	**Hexanal**	HS	29,88	52,34	0	0	220,64	154,28
		U	0,62	1,76	0	0	7,1	6,55
**35**	**N-Benzyl-N-ethyl-p-isopropylbenzamide**	B	24,78	95,50	0	0	504,85	326,23
**36**	**n-Hexane**	HS	158,27	371,24	0	0	1467,4	1304,08
		B	115,33	190,06	28,01	0	850,54	729,95
		U	4,01	5,86	2,81	0	25,38	22,68
**37**	**Octane**	B	13,79	39,44	0	0	221,4	107,77
**38**	**Oxime-, methoxy-phenyl-**	HS	14,31	31,61	0	0	110	91,49

		B	6,13	15,54	0	0	91,55	29,06
		U	11,96	46,75	0	0	225,33	184,06
**39**	**Pentanal**	HS	17,17	36,06	0	0	139,16	116,12
		B	6,29	16,05	0	0	90,5	34,41
**40**	**Pentane**	B	113,08	244,68	0	0	1086,09	853,25
**41**	**Propanal, 2-methyl-**	U	1,99	6,46	0	0	29,62	25,44
**42**	**Propane, 2-(ethenyloxy)-**	HS	6,06	19,62	0	0	78,12	66,81
		B	2,72	7,70	0	0	35,09	26,51
		U	2,65	5,21	0	0	16,05	15,67
**43**	**Pyrrole**	HS	9,71	36,95	0	0	198,28	102,86
		U	2,44	6,28	0	0	24,29	21,87

Table S2 Characteristics of VOC predictors. For each VOC it's reported its CAS number, CID in PubChem [50], code in CompTox Dashboard (EPA) [48], ID in Human Metabolome Database HMDB [46] and its presence in Comparative Toxicogenomics Database [49]. The column Biofluids (HS, B, U) reports in which biofluids the VOC was found. The Spermiogram Parameter Prediction reports for all the spermiogram parameters the label: Biofluid/Datasets that refers to the origin biofluid/dataset of the regression model where the specific VOC contributed as a predictive variable.

								Spermiogram Parameter Prediction Label: Biofluid/Datasets								
n.	VOC predictor	CAS	PubChem CID [a]	CompTox Dashboard (EPA) [b]	Human Metabolome Database HMDB ID [c]	Comparative Toxicogenomics Database [d]	Biofluids (HS, B, U)	Cells conc.	Progressive motility	Total motility	Immotiles	Morph. Anomalies	Head Anomalies	Neck Anomalies	Tail Anomalies	Round cells
1	(3-Methoxy-phenyl)-(6-methyl-4-phenyl-quinazolin-2-yl)-amine	1000317-62-7	/	/	/	/	HS; B	B/B and HS/HS+B and B/HS+B	B/B and HS/HS+B and B/HS+B	HS/HS+B and B/HS+B	HS/HS+B and B/HS+B		B/B and HS/HS and HS/HS+B	HS/HS	B/B and B/HS+B	B/B
2	1-(6-Methyl-benzothiazol-2-yl)-3-(4-methyl-benzoyl)-thiourea	131120-14-4	/	/	/	/	HS; B		B/B and HS/HS+B and B/HS+B			B/B+U	B/B and B/HS+B	B/B and HS/HS+B	B/B and HS/HS+B and B/HS+B	B/B+U and HS/HS and B/HS+B+U
3	11H-Dibenzo[b,e][1,4]diazepin-11-one, 5,10-dihydro-5-[3-(methylamino)propyl]	13450-70-9	45221	DTXSID20158754	/	X	B; U	B/B+U and B/HS+B and B/HS+B+U	B/HS+B and B/HS+B+U	U/U and B/HS+B and B/HS+B+U	B/HS+B and B/HS+B+U	B/B and U/U	U/U and U/HS+U	B/B	B/B and B/HS+B and U/B+U and U/HS+B+U	U/U and B/HS+B
4	1-Anthracenamine	610-49-1	11885	DTXSID00209859	HMDB0243820	X	HS	HS/HS+B and HS/HS+U and HS/HS+B+U	HS/HS+B and HS/HS+B+U	HS/HS+B and HS/HS+U and	HS/HS+B and HS/HS+B+U		HS/HS and HS/HS+B		HS/HS+B	HS/HS+B
5	1H-Indole, 5-methyl-2-phenyl-	13228-36-9	83247	DTXSID9074692	HMDB0000466	/	U		U/U and U/B+U and U/HS+U and U/HS+B+U	U/U and U/B+U and U/HS+U and U/HS+B+U	U/U and U/B+U and U/HS+B+U	U/U				U/U and U/HS+U
6	2,4,5-Trioxoimidazolidine	120-89-8	67126	DTXSID2059516	HMDB0062802	X	U		U/U	U/U and HS/HS+U	U/U	U/U	U/U	U/U and U/HS+B+U	U/B+U	U/U
7	2-Anthracenamine	613-13-8	11937	DTXSID2024458	HMDB0245007	X	HS; B; U			U/U and B/B and U/HS+U	U/U and U/B+U	U/U and U/B+U and B/HS+B	U/U and B/B and B/HS+B	U/U and B/B and B/HS+B	HS/HS+B and B/HS+B	U/U and B/HS+B
8	2-Butanone	78-93-3	6569	DTXSID3021516	HMDB0000474	X	U	U/U and U/HS+B+U				U/U		U/U		
9	2-Chloro-4-(4-methoxyphenyl)-6-(4-nitrophenyl)pyrimidine	63673-76-7	631100	/	/	/	B	B/B+U and B/B	B/B and B/HS+B and B/HS+B+U	B/B and B/B+U and B/HS+B and B/HS+B+U	B/B and B/HS+B and B/HS+B+U					B/B+U
10	2-Ethyl-oxetane	1000386-40-2	521218	/	/	/	HS; B; U	U/B+U and B/B and HS/HS+B+U	B/B	B/B	U/U and B/B and B/B+U	U/U and B/B+U and B/B	U/U and U/B+U and HS/HS	B/B and B/HS+B+U	B/B+U and B/B and HS/HS+B and B/HS+B and B/HS+B+U	B/B and U/HS+U and U/B+U
11	2H-Pyrrol-2-one, 1,5-dihydro-1-(4-methoxyphenyl)-5,5-diphenyl-	53774-23-5	631062	/	/	/	B; U	U/B+U	B/B	B/B	B/B	U/U and B/B+U and B/B	U/U		B/HS+B	U/U and U/B+U and U/HS+B+U

12	**2-Pentanone**	107-87-9	7895	DTXSID0021888	HMDB0034235	X	B; U	U/U and U/B+U and B/B and U/HS+U and U/HS+B+U	U/B+U and B/HS+B	B/B and B/HS+B	U/U and B/B and U/B+U and B/HS+B	B/HS+B and U/HS+U and U/HS+B+U	B/B+U and U/HS+U	B/B and U/B+U	B/B	U/U and B/B and U/HS+U and U/B+U
13	**3-Aminopyrrolidine**	79286-79-6	164401	DTXSID70276538	/	/	HS; B; U	U/U and B/B+U and HS/HS and HS/HS+B and B/HS+B and HS/HS+U and U/HS+U and HS/HS+B+U	U/U and B/B and U/B+U and HS/HS+B and B/HS+B and U/HS+U	U/U and B/B and HS/HS+B and B/HS+B and HS/HS+U	U/U and B/B and HS/HS+B and B/HS+B	U/U	U/U	U/U and B/B and HS/HS+B	B/B	B/B and HS/HS and B/HS+B and B/HS+B+U
14	**3-Hexanone**	589-38-8	11509	DTXSID2021608	HMDB0000753	X	U	U/B+U	U/U		U/U	U/HS+B+U and U/HS+B+U	U/U and U/B+U and U/HS+B+U	U/U		U/U
15	**4-(4-Chlorophenyl)-2,6-diphenylpyridine**	1498-82-4	631072	DTXSID20348153	/	/	HS; B;U	B/B+U and B/B		U/U and B/B and B/B+U and HS/HS+U		B/B+U	B/B and HS/HS and U/B+U and HS/HS+B and B/HS+B and	B/B+U and HS/HS and HS/HS+B and B/HS+B	B/B and HS/HS and HS/HS+B and B/HS+B	B/B and HS/HS+B
16	**4-Heptanone**	123-19-3	31246	DTXSID6047650	HMDB0004814	X	U			U/U	U/U and U/B+U			U/U and U/HS+U and U/HS+B+U	U/HS+U	
17	**5,9-Dodecadien-2-one, 6,10-dimethyl-, (E,E))-**	13125-74-1	114621	DTXSID5065358	/	/	U	U/U	U/U	U/U	U/U		U/B+U		U/B+U	U/U
18	**Acetic acid, sodium salt**	127-09-3	517045	DTXSID2027044	/	/	HS; B; U	B/B	B/B and B/HS+B and HS/HS+B+U	B/B and B/B+U and B/HS+B and B/HS+B+U	U/U and B/B and B/B+U and B/HS+B and B/HS+B+U	B/HS+B	HS/HS and U/B+U	U/U and U/B+U and B/HS+B and HS/HS+U and HS+B+U	HS/HS and HS/HS+B and HS/HS+U and HS/HS+B+U	
19	**Acetone**	67-64-1	180	DTXSID8021482	HMDB01659	X	HS; B; U	U/B+U and B/B and HS/HS and U/HS+U and U/HS+B+U		B/B	B/B	HS/HS+B	U/U and B/HS+B	U/U and HS/HS and HS/HS+B	B/B and HS/HS and B/HS+B	U/U and HS/HS and HS/HS+B+U
20	**alpha-Pinene**	80-56-8	6654	DTXSID4026501	HMDB0006525	X	B		B/B		B/B+U			B/B and B/HS+B+U	B/B+U and B/HS+B+U	
21	**Auramine**	492-80-8	10298	DTXSID7043821	HMDB0248724	X	HS; B	B/B+U and B/B and B/HS+B	B/B and B/HS+B	B/B	B/B		HS/HS and HS/HS+B and B/HS+B and HS/HS+U and HS/HS+B+U		B/B and HS/HS and HS/HS+B	HS/HS and HS/HS+B and B/HS+B
22	**Benzaldehyde, 2-nitro-, diaminomethylidenhydrazone**	102632-31-5	1E+07	DTXSID20879432	/	/	B; U			B/B+U	B/B+U		B/HS+B		U/B+U	U/U and B/B

**23**	**Butanal**	123-72-8	261	DTXSID8021513	HMDB0003543	X	HS; U	U/U and U/HS+U	U/HS+U and U/HS+B+U	U/B+U and U/HS+U and U/HS+B+U	U/HS/HS+U	U/U	U/U and HS/HS and HS/HS+B	U/U and U/B+U and U/HS+B+U	HS/HS and HS/HS+B and HS/HS+B+U	
**24**	**Butanal, 2-methyl**	96-17-3	7284	DTXSID2021818	HMDB0031526	/	HS	HS/HS+U	HS/HS+U and HS/HS+B+U	HS/HS+B+U	HS/HS+B+U	HS/HS	HS/HS and HS/HS+U	HS/HS+U and HS/HS+B+U	HS/HS	HS/HS and HS/HS+B
**25**	**Butanal, 3-methyl-**	590-86-3	11552	DTXSID1021619	HMDB0006478	X	HS; B; U	B/B and B/HS+B	U/U and B/B and B/HS+B and HS/HS+U	B/B	U/U and B/B and U/B+U	HS/HS	U/U and HS/HS+U	U/U and B/B and B/HS+B and HS/HS+B	B/B	U/U and B/B and HS/HS
**26**	**Butane, 2-methyl-**	78-78-4	6556	DTXSID8025468	HMDB0253668	X	HS; B	HS/HS+U	B/B and HS/HS+B and B/HS+B	B/HS+B and HS/HS+U	B/HS+B	HS/HS and HS/HS+B	B/B and HS/HS and HS/HS+B	HS/HS+B	HS/HS and HS/HS+B	HS/HS
**27**	**Cyclohexane**	110-82-7	8078	DTXSID4021923	HMDB0029597	/	B	B/B		B/B	B/B	B/B+U and B/B	B/B and B/HS+B and B/HS+B+U	B/B and B/HS+B		B/B
**28**	**Cyclopentane, methyl-**	96-37-7	7296	DTXSID3025590	HMDB0031542	X	B	B/B	B/HS+B	B/HS+B	B/B and B/HS+B	B/B	B/B and B/HS+B	B/B	B/HS+B	
**29**	**Disulfide, dimethyl**	624-92-0	12232	DTXSID4025117	HMDB0005879	X	U			U/U and U/HS+U	U/U			U/B+U and U/HS+B+U	U/B+U	U/U and U/HS+U
**30**	**D-Limonene**	5989-27-5	440917	DTXSID1020778	HMDB0003375	X	HS; B	B/B and HS/HS+B	B/B	B/B	B/B	B/HS+B and HS/HS+U	HS/HS and HS/HS+U		HS/HS	HS/HS
**31**	**Fluoren-9-ol, 3,6-dimethoxy-9-(2-phenylethynyl)-**	1000217-31-2	631096	/	/	/	HS; U	U/U and U/B+U and U/HS+U	U/U and U/HS+B+U	U/U and U/B+U and U/HS+B+U	U/U and U/HS+B+U	U/B+U	U/U and HS/HS	U/U and HS/HS+B	HS/HS	U/U
**32**	**Heptanal**	111-71-7	8130	DTXSID0021597	HMDB0031475	X	B	B/B and B/HS+B		B/B	B/B	B/B	B/B and B/B+U and B/HS+B	B/B	B/B+U and B/B and B/HS+B	B/B
**33**	**Heptane**	142-82-5	8900	DTXSID6024127	HMDB0031447	X	B	B/B and B/HS+B		B/B and B/HS+B	B/B and B/HS+B	B/HS+B	B/HS+B			B/B+U
**34**	**Hexanal**	66-25-1	6184	DTXSID2021604	HMDB0005994	X	HS; U	HS/HS+B	HS/HS+B+U	U/U and U/HS+U	U/U	U/B+U	U/U and U/B+U and HS/HS and HS/HS+U and HS/HS+B+U	U/U and HS/HS		U/U and HS/HS and HS/HS+U
**35**	**N-Benzyl-N-ethyl-p-isopropylbenzamide**	015089-22-2	590384	/	/	/	B	B/B				B/B		B/B	B/B and B/HS+B	
**36**	**n-Hexane**	110-54-3	8058	DTXSID0021917	HMDB0029600	X	HS; B; U	HS/HS+B and B/HS+B	HS/HS+B and B/HS+B	U/U and B/B and HS/HS+B and B/HS+B and HS/HS+B+U		HS/HS and HS/HS+B and HS/HS+B+U	U/U and B/B and B/HS+B and B/HS+B	U/U	B/B and HS/HS and HS/HS+B and B/HS+B and U/B+U and U/HS+B+U	B/B and B/B+U and HS/HS+B and HS/HS+U and HS/HS+B+U

37	**Octane**	111-65-9	356	DTXSID0026882	HMDB0001485	X	B	B/B+U and B/B and B/HS+B	B/B and B/HS+B							B+B+U and B/HS+B+U
38	**Oxime-, methoxy-phenyl-**	1000222-86-6	1E+07	/	/	X	HS; U			U/U		U/B+U	U/U	HS/HS+B	HS/HS and HS/HS+B+U	U/U and HS/HS+U
39	**Pentanal**	110-62-3	8063	DTXSID7021653	HMDB0031206	X	HS; B	B/B	B/B	B/B	B/B	HS/HS and B/HS+B			HS/HS and HS/HS+B+U	B/B and HS/HS and HS/HS+B and B/HS+B and HS/HS+U and
40	**Pentane**	109-66-0	8003	DTXSID2025846	HMDB0029603	X	B	B/HS+B	B/B	B/B	B/B		B/HS+B and B/HS+B+U			
41	**Propanal, 2-methyl-**	78-84-2	6561	DTXSID9021635	HMDB0031243	X	U	U/B+U		U/U and U/B+U	U/B+U			U/U		U/U
42	**Propane, 2-(ethenyloxy)-**	926-65-8	13557	DTXSID0061292	/	/	HS; B; U	U/U and B/B and HS/HS+B+U				U/B+U and U/HS+U and B/HS+B+U and U/HS+B+U	U/U and HS/HS+B and B/HS+B	B/B+U and HS/HS and HS/HS+B	B/B+U and B/HS+B and B/HS+B+U	U/U and B/B and U/B+U and HS/HS and U/HS+U and U/HS+B+U
43	**Pyrrole**	109-97-7	8027	DTXSID5021910	HMDB35924	/	HS; U		U/U and U/B+U		U/B+U	U/U and HS/HS and HS/HS+B and HS/HS+U	U/U		U/B+U	U/U and HS/HS

[a] PubChem Compound, website https://pubchem.ncbi.nlm.nih.gov/ ([50])

[b] CompTox Chemicals Dashboard (EPA), website https://comptox.epa.gov/dashboard/ ([48])

[c] HMDB The Human Metabolome Database, website https://hmdb.ca/ ([46])

[d] CTDbase Comparative Toxicogenomics Database, website https://ctdbase.org/ ([49])

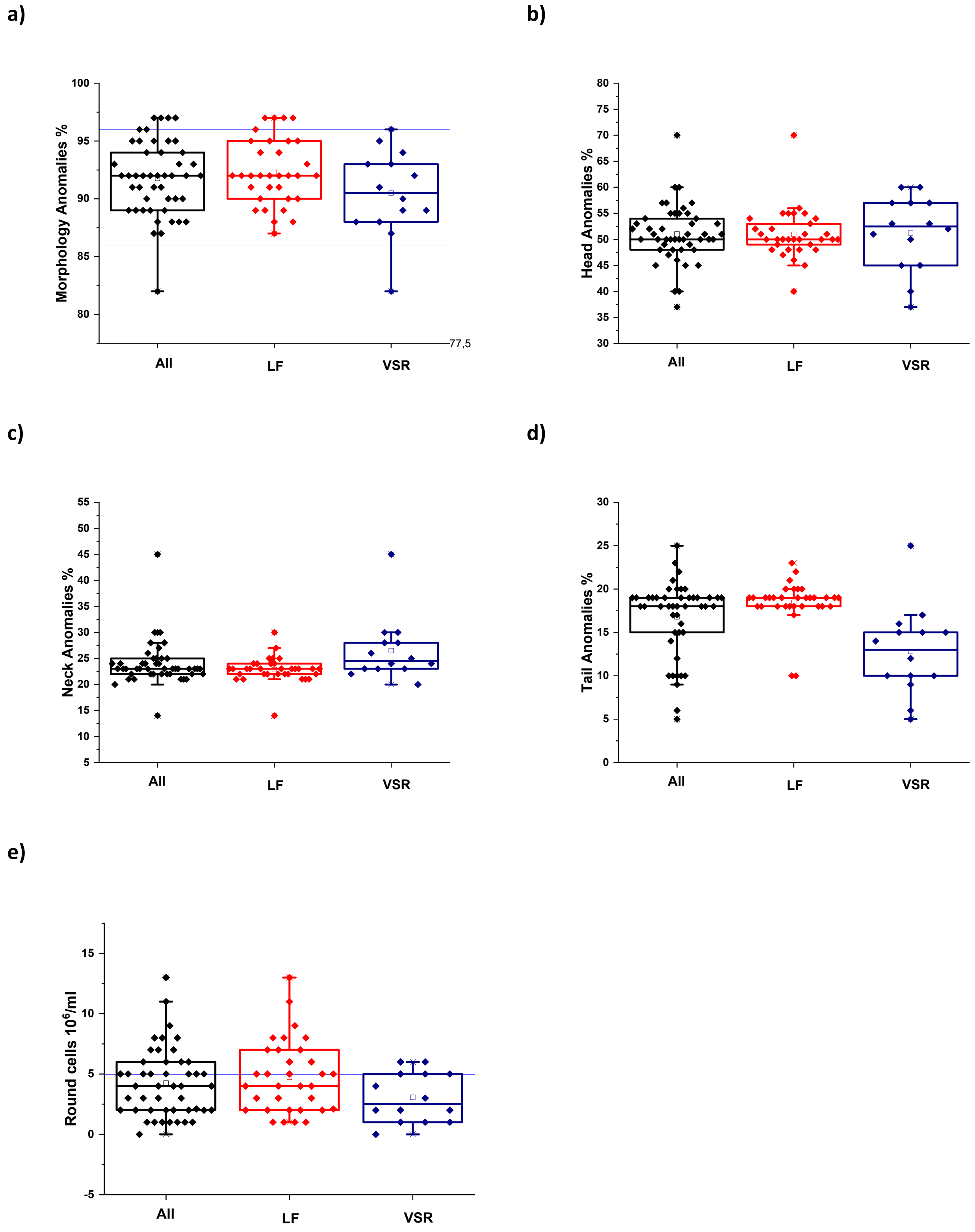

Figure S1 Box & whiskers plot displaying the dispersion of the seminogram parameters related to sperm morphology and round cells: a) total morphology anomalies; b) head anomalies; c) neck anomalies; d) tail anomalies; e) round cells concentration, in the subgroups of population living in *Land of Fire*s (LF) and in *Valley of Sacco river* (VSR) and in the total population (all=LF+VSR).

a)

Morphology Anomalies %	Multiple R	Multiple R^2	Adjusted R^2	SS Model	df Model	MS Model	SS Residual	df Residual	MS Residual	F	p
HS + B + U	0,951849	0,906016	0,847275	137,26	5	27,452	14,239	8	1,7798	15,424110	0,000626
HS + B	0,892523	0,796597	0,669470	208,24	10	20,824	53,171	16	3,3232	6,266150	0,000656
HS + U	0,859512	0,738761	0,651681	113,86	4	28,464	40,262	12	3,3551	8,483729	0,001727
B + U	0,977555	0,955614	0,892205	186,93	10	18,693	8,6824	7	1,24035	15,07068	0,000805
HS	0,732860	0,537084	0,430257	144,459	6	24,077	124,51	26	4,7889	5,027608	0,001534
B	0,662527	0,438943	0,312252	197,28	7	28,182	252,159	31	8,1342	3,464687	0,007413
U	0,957156	0,916148	0,846271	182,91	10	18,291	16,741	12	1,3951	13,110940	0,000054

b)

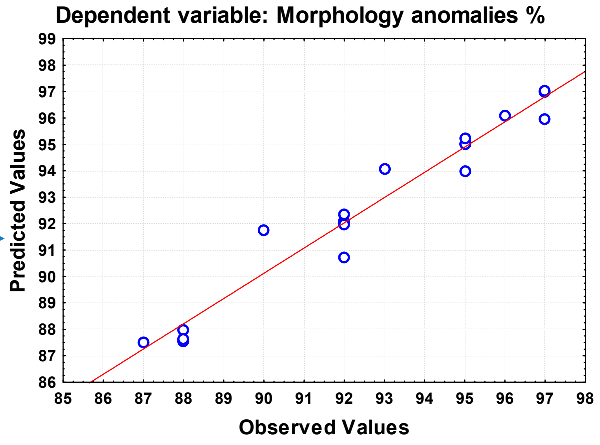

c) VOC predictors

1) Propane, 2-(ethenyloxy)-_U
2) Oxime-, methoxy-phenyl-__U
3) 2-Ethyl-oxetane_B
4) Fluoren-9-ol, 3,6-dimethoxy-9-(2-phenylethynyl)-_U
5) Hexanal_U
6) 4-(4-Chlorophenyl)-2,6-diphenylpyridine_B
7) 1-(6-Methyl-benzothiazol-2-yl)-3-(4-methyl-benzoyl)-thiourea_B
8) Cyclohexane_B
9) 2-Anthracenamine_U
10) 2H-Pyrrol-2-one, 1,5-dihydro-1-(4-methoxyphenyl)-5,5-diphenyl-_B

Figure S2 a) Overall fit of the MLR model described by the test of SS Whole Model vs. SS Residual for sperm **morphology anomalies** (in percentage); b) Observed vs. Predicted values for sperm morphology anomalies as result of the MLR analysis for the data group U based on selected VOC predictors; c) pattern of VOCs used as predictor variables in MLR model as selected by Pareto chart; the order in the list reflects the greatest predictive contribution to the model (VOC name_X; X= B blood or U urine or HS human semen). Notation for numeric values: comma "," is the decimal separator (SI).

a)

Head Anomalies%	Multiple R	Multiple R^2	Adjusted R^2	SS Model	df Model	MS Model	SS Residual	df Residual	MS Residual	F	p
HS + B + U	0,986646	0,973470	0,950730	230,92	6	38,487	6,293	7	0,8990	42,808650	0,000036
HS + B	0,999603	0,999206	0,994842	743,71	22	33,805	0,591	4	0,1477	228,939800	0,000041
HS + U	0,954244	0,910582	0,841034	218,43	7	31,205	21,450	9	2,3833	13,092920	0,000458
B + U	0,950425	0,903307	0,794527	298,94	9	33,216	32,0000	8	4,00000	8,30401	0,003320
HS	0,869429	0,755907	0,609451	622,501	12	51,875	201,01	20	10,0507	5,161331	0,000648
B	0,730072	0,533006	0,388076	726,80	9	80,756	636,789	29	21,9582	3,677693	0,003573
U	0,995314	0,990650	0,965718	342,68	16	21,417	3,234	6	0,5390	39,733730	0,000093

b)

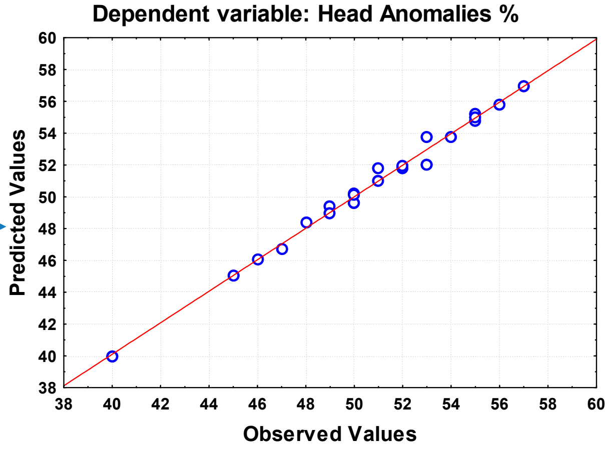

c) VOC predictors

1) Oxime-, methoxy-phenyl-__U
2) 11H-Dibenzo[b,e][1,4]diazepin-11-one, 5,10-dihydro -5-[3-(methylamino)propyl]-_U
3) 3-Hexanone_U
4) 2H-Pyrrol-2-one, 1,5-dihydro-1-(4-methoxyphenyl)-5,5-diphenyl-_U
5) Propane, 2-(ethenyloxy)-_U
6) Acetone_U
7) Hexanal_U
8) 2-Ethyl-oxetane_U
9) n-Hexane_U
10) 3-Aminopyrrolidine_U
11) Butanal_U
12) 2-Anthracenamine_U
13) Butanal, 3-methyl-_U
14) Pyrrole_U
15) 2,4,5-Trioxoimidazolidine_U
16) Fluoren-9-ol, 3,6-dimethoxy-9-(2-phenylethynyl)-_U

Figure S3 a) Overall fit of the MLR model described by the test of SS Whole Model vs. SS Residual for sperm **head anomalies** (in percentage); b) Observed vs. Predicted values for sperm head anomalies as result of the MLR analysis for the data group U based on selected VOC predictors; c) pattern of VOCs used as predictor variables in MLR model as selected by Pareto chart; the order in the list reflects the greatest predictive contribution to the model (VOC name_X; X= B blood or U urine or HS human semen). Notation for numeric values: comma "," is the decimal separator (SI).

a)

Neck Anomalies %	Multiple R	Multiple R^2	Adjusted R^2	SS Model	df Model	MS Model	SS Residual	df Residual	MS Residual	F	p
HS + B + U	0,997846	0,995696	0,988809	130,65	8	16,331	0,565	5	0,1130	144,578500	0,000017
HS + B	0,907557	0,823660	0,617931	208,36	14	14,883	44,607	12	3,7173	4,003611	0,010501
HS + U	0,902950	0,815320	0,769149	110,68	3	36,893	25,070	12	2,0892	17,659040	0,000107
B + U	0,813958	0,662528	0,478452	98,35	6	16,391	50,0959	11	4,55417	3,59921	0,031759
HS	0,761019	0,579150	0,461312	147,525	7	21,075	107,20	25	4,2881	4,914799	0,001346
B	0,721634	0,520755	0,299565	414,47	12	34,539	381,430	26	14,6704	2,354334	0,032890
U	0,985085	0,970392	0,918578	151,30	14	10,807	4,616	8	0,5770	18,728430	0,000140

b)

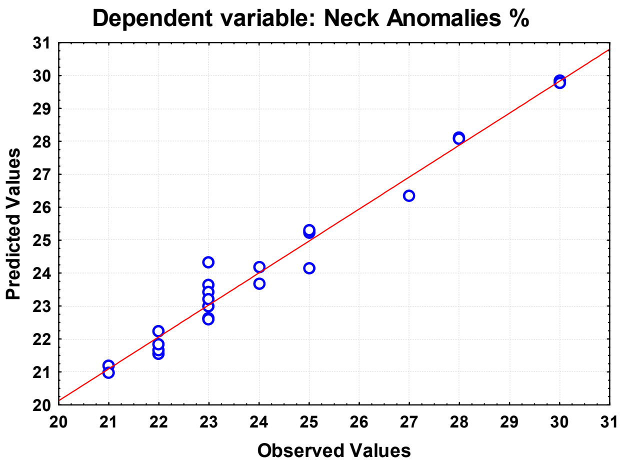

c) VOC predictors

1) Butanal_U
2) 3-Aminopyrrolidine_U
3) Acetic acid, sodium salt_U
4) 2,4,5-Trioxoimidazolidine_U
5) n-Hexane_U
6) 2-Butanone_U
7) 4-Heptanone_U
8) Acetone_U
9) Hexanal_U
10) 2-Anthracenamine_U
11) Propanal, 2-methyl-_U
12) 3-Hexanone_U
13) Butanal, 3-methyl-_U
14) Fluoren-9-ol, 3,6-dimethoxy-9-(2-phenylethynyl)-_U

Figure S4 a) Overall fit of the MLR model described by the test of SS Whole Model vs. SS Residual for sperm **neck anomalies** (in percentage); b) Observed vs. Predicted values for sperm neck anomalies as result of the MLR analysis for the data group U based on selected VOC predictors; c) pattern of VOCs used as predictor variables in MLR model as selected by Pareto chart; the order in the list reflects the greatest predictive contribution to the model (VOC name_X; X= B blood or U urine or HS human semen). Notation for numeric values: comma "," is the decimal separator (SI).

a)

Tail Anomalies %	Multiple R	Multiple R^2	Adjusted R^2	SS Model	df Model	MS Model	SS Residual	df Residual	MS Residual	F	p
HS + B + U	0,999462	0,998925	0,993012	4331,05	11	393,732	4,661	2	2,3307	168,935000	0,005899
HS + B	0,999772	0,999545	0,996056	496,44	23	21,584	0,226	3	0,0753	286,495900	0,000293
HS + U	0,608807	0,370646	0,280738	106,70	2	53,351	181,180	14	12,9414	4,122518	0,039108
B + U	0,946014	0,894943	0,821404	275,64	7	39,378	32,3574	10	3,23574	12,16954	0,000358
HS	0,862206	0,743399	0,567830	411,077	13	31,621	141,89	19	7,4680	4,234225	0,002315
B	0,910540	0,829082	0,740205	682,82	13	52,525	140,766	25	5,6306	9,328398	0,000001
U	0,629402	0,396146	0,261957	126,46	4	31,614	192,761	18	10,7089	2,952139	0,048737

b)

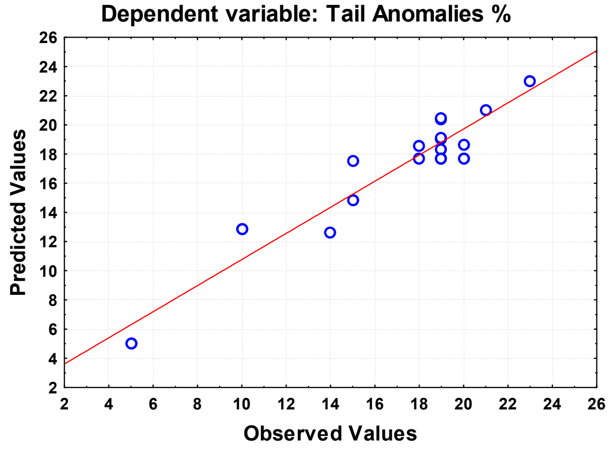

c) VOC predictors

1) Benzaldehyde, 2-nitro-, diaminomethylidenhydrazone_U
2) Disulfide, dimethyl_U
3) 2-Ethyl-oxetane_B
4) Heptanal_B
5) Propane, 2-(ethenyloxy)-_B
6) .alpha.-Pinene_B
7) 5,9-Dodecadien-2-one, 6,10-dimethyl-, (E,E))-_U

Figure S5 a) Overall fit of the MLR model described by the test of SS Whole Model vs. SS Residual for **sperm tail anomalies** (in percentage); b) Observed vs. Predicted values for sperm tail anomalies as result of the MLR analysis for the data group U based on selected VOC predictors; c) pattern of VOCs used as predictor variables in MLR model as selected by Pareto chart; the order in the list reflects the greatest predictive contribution to the model (VOC name_X; X= B blood or U urine or HS human semen). Notation for numeric values: comma "," is the decimal separator (SI).

a)

Round cells 10^6/ml	Multiple R	Multiple R^2	Adjusted R^2	SS Model	df Model	MS Model	SS Residual	df Residual	MS Residual	F	p
HS + B + U	0,975114	0,950847	0,872202	142,15	8	17,769	7,348	5	1,4697	12,090350	0,006950
HS + B	0,918359	0,843384	0,728532	162,86	11	14,805	30,242	15	2,0162	7,343238	0,000306
HS + U	0,988538	0,977206	0,947901	192,57	9	21,396	4,492	7	0,6417	33,345000	0,000063
B + U	0,960418	0,922403	0,835106	192,12	9	21,346	16,1618	8	2,02022	10,56628	0,001458
HS	0,895012	0,801047	0,681675	202,624	12	16,885	50,33	20	2,5163	6,710513	0,000108
B	0,796776	0,634852	0,466323	174,97	12	14,581	100,638	26	3,8707	3,767008	0,002249
U	0,997482	0,994971	0,972338	272,45	18	15,136	1,377	4	0,3443	43,961700	0,001108

b)

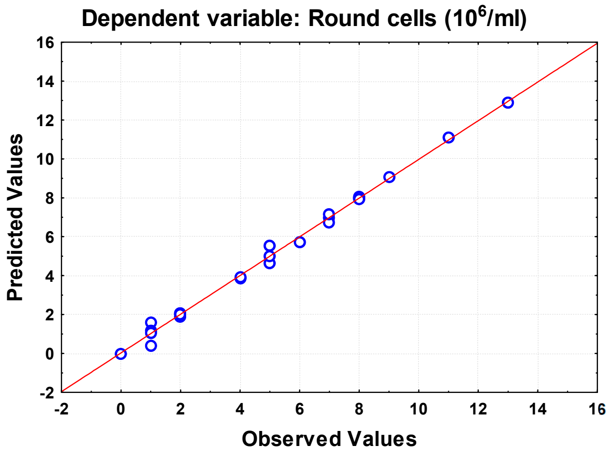

c) VOC predictors

1) 1H-Indole, 5-methyl-2-phenyl-_U
2) Propane, 2-(ethenyloxy)-_U
3) 2-Anthracenamine_U
4) Hexanal_U
5) Disulfide, dimethyl_U
6) 2-Pentanone_U
7) 11H-Dibenzo[b,e][1,4]diazepin-11-one, 5,10-dihydro -5-[3-(methylamino)propyl]-_U
8) 3-Hexanone_U
9) Pyrrole_U
10) Benzaldehyde, 2-nitro-, diaminomethylidenhydrazone_U
11) 5,9-Dodecadien-2-one, 6,10-dimethyl-, (E,E))-_U
12) Propanal, 2-methyl-_U
13) Acetone_U
14) Oxime-, methoxy-phenyl-__U
15) Fluoren-9-ol, 3,6-dimethoxy-9-(2-phenylethynyl)-_U
16) 2,4,5-Trioxoimidazolidine_U
17) 2H-Pyrrol-2-one, 1,5-dihydro-1-(4-methoxyphenyl)-5,5-diphenyl-_U

Figure S6 a) Overall fit of the MLR model described by the test of SS Whole Model vs. SS Residual for **round cells** (in percentage); b) Observed vs. Predicted values for round cells as result of the MLR analysis for the data group U based on selected VOC predictors; c) pattern of VOCs used as predictor variables in MLR model as selected by Pareto chart; the order in the list reflects the greatest predictive contribution to the model (VOC name_X; X= B blood or U urine or HS human semen). Notation for numeric values: comma "," is the decimal separator (SI).

a)

Concentration (10^6 cells/ml)	Multiple R	Multiple R^2	Adjusted R^2	SS Model	df Model	MS Model	SS Residual	df Residual	MS Residual	F	p
HS + B + U	0,988824	0,977774	0,927764	12898,02	9	1433,113	293,194	4	73,2984	19,551760	0,005803
HS + B	0,970233	0,941352	0,847516	24610,99	16	1538,187	1533,303	10	153,3303	10,031850	0,000388
HS + U	0,969477	0,939886	0,862596	15282,99	9	1698,110	977,484	7	139,6405	12,160580	0,001679
B + U	0,989074	0,978268	0,907639	15121,03	13	1163,156	335,9117	4	83,97793	13,85074	0,010628
HS	0,495168	0,245191	0,194870	7428,854	2	3714,427	22869,39	30	762,3130	4,872575	0,014708
B	0,928148	0,861459	0,736772	29565,19	18	1642,510	4754,710	20	237,7355	6,908982	0,000039
U	0,880287	0,774905	0,669861	15263,00	7	2180,429	4433,608	15	295,5738	7,376934	0,000622

b)

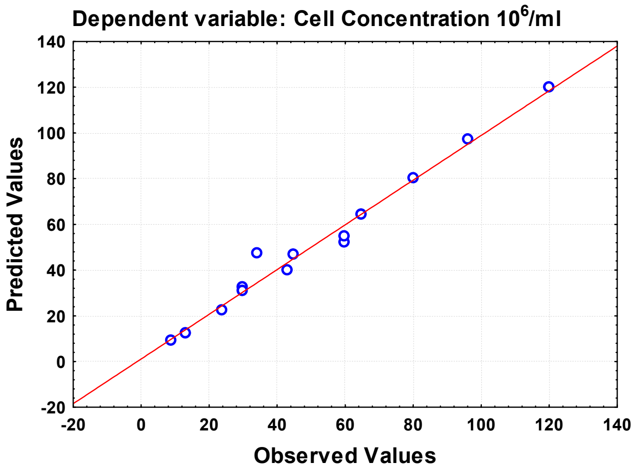

c) VOC predictors

1) 11H-Dibenzo[b,e][1,4]diazepin-11-one, 5,10-dihydro-5-[3-(methylamino)propyl]-_B
2) 1-Anthracenamine_HS
3) 2-Pentanone_U
4) Propane, 2-(ethenyloxy)_HS
5) 2-Butanone_U
6) 3-Aminopyrrolidine_HS
7) 2-Ethyl-oxetane_HS
8) Acetone_U

Figure S7 a) Overall fit of the MLR model described by the test of SS Whole Model vs. SS Residual for **sperm concentration** (in 10^6/ml); b) Observed vs. Predicted values for sperm concentration as result of the MLR analysis for the data group HS+B+U based on selected VOC predictors; c) pattern of VOCs used as predictor variables in MLR model as selected by Pareto chart; the order in the list reflects the greatest predictive contribution to the model (VOC name_X; X= B blood or U urine or HS human semen). Notation for numeric values: comma "," is the decimal separator (SI).

a)

Progressive motility %	Multiple R	Multiple R^2	Adjusted R^2	SS Model	df Model	MS Model	SS Residual	df Residual	MS Residual	F	p
**HS + B + U**	0,998162	0,996328	0,988065	4319,79	9	479,977	15,921	4	3,9804	120,586400	0,000165
**HS + B**	0,994035	0,988107	0,948462	25833,35	20	1291,667	310,947	6	51,8245	24,923900	0,000342
**HS + U**	0,949293	0,901157	0,856229	4304,35	5	860,870	472,119	11	42,9199	20,057590	0,000034
**B + U**	0,833066	0,694000	0,599846	4148,58	4	1037,144	1829,2020	13	140,70780	7,37091	0,002503
**HS**	/	/	/	/	/	/	/	/	/	/	/
**B**	0,900439	0,810791	0,687393	10912,41	15	727,494	2546,562	23	110,7201	6,570570	0,000035
**U**	0,914171	0,835708	0,741827	4957,929000	8,000000	619,741100	974,679900	14,000000	69,619990	8,901769	0,000244

b)

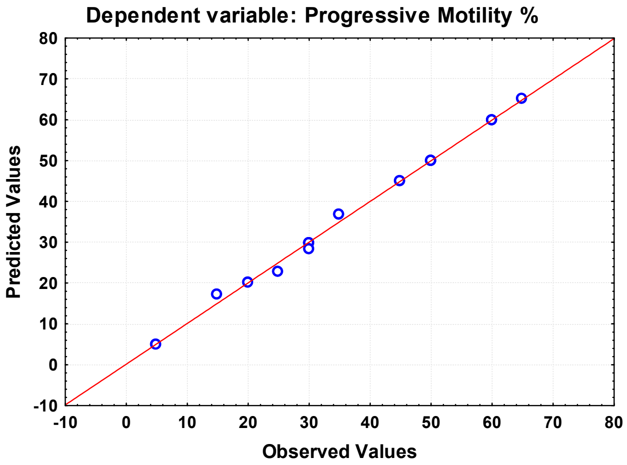

c) VOC predictors

1) 1H-Indole, 5-methyl-2-phenyl-_U
2) 11H-Dibenzo[b,e][1,4]diazepin-11-one, 5,10-dihydro-5-[3-(methylamino)propyl]-_B
3) Fluoren-9-ol, 3,6-dimethoxy-9-(2-phenylethynyl)-_U
4) Butanal_U
5) Acetic acid, sodium salt_HS
6) Hexanal_HS
7) 2-Chloro-4-(4-methoxyphenyl)-6-(4-nitrophenyl)pyrimidine_B
8) Butanal, 2-methyl-_HS
9) 1-Anthracenamine_HS

Figure S8 a) Overall fit of the MLR model described by the test of SS Whole Model vs. SS Residual for sperm **progressive motility** (in percentage); b) Observed vs. Predicted values for sperm progressive motility as result of the MLR analysis for the data group HS+B+U based on selected VOC predictors; c) pattern of VOCs used as predictor variables in MLR model as selected by Pareto chart; the order in the list reflects the greatest predictive contribution to the model (VOC name_X; X= B blood or U urine or HS human semen). Notation for numeric values: comma "," is the decimal separator (SI).

a)

Total Motility %	Multiple R	Multiple R^2	Adjusted R^2	SS Model	df Model	MS Model	SS Residual	df Residual	MS Residual	F	p
**HS + B + U**	0,998866	0,997733	0,992633	5368,16	9	596,462	12,196	4	3,0491	195,619100	0,000063
**HS + B**	0,974892	0,950414	0,892564	10746,72	14	767,623	560,689	12	46,7241	16,428840	0,000011
**HS + U**	0,982579	0,965462	0,907900	6761,08	10	676,108	241,865	6	40,3108	16,772360	0,001299
**B + U**	0,970786	0,942426	0,891250	6184,67	8	773,084	377,8270	9	41,98078	18,41519	0,000102
**HS**	/	/	/	/	/	/	/	/	/	/	/
**B**	0,944713	0,892483	0,795717	17751,25	18	986,181	2138,491	20	106,9246	9,223145	0,000004
**U**	0,985701	0,971606	0,921917	7870,01	14	562,144	229,990	8	28,7487	19,553710	0,000119

b)

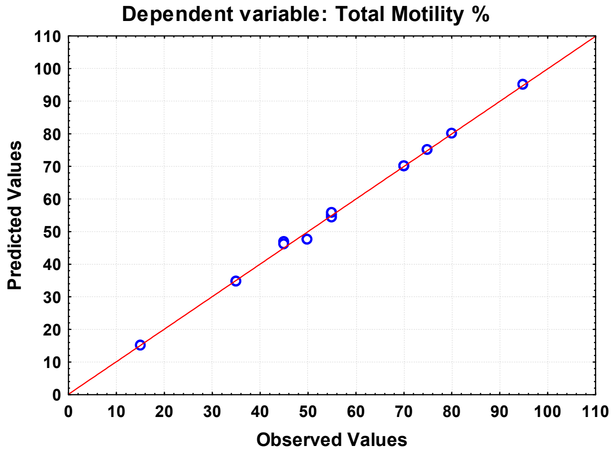

c) VOC Predictors

1) 1-Anthracenamine_HS
2) 11H-Dibenzo[b,e][1,4]diazepin-11-one, 5,10-dihydro-5-[3-(methylamino)propyl]-_B
3) Fluoren-9-ol, 3,6-dimethoxy-9-(2-phenylethynyl)-_U
4) 1H-Indole, 5-methyl-2-phenyl-_U
5) Acetic acid, sodium salt_B
6) 2-Chloro-4-(4-methoxyphenyl)-6-(4-nitrophenyl)pyrimidine_B
7) n-Hexane_HS
8) Butanal, 2-methyl-_HS
9) Butanal_U

Figure S9 a) Overall fit of the MLR model described by the test of SS Whole Model vs. SS Residual for **sperm total motility** (in percentage); b) Observed vs. Predicted values for sperm total motility as result of the MLR analysis for the data group HS+B+U based on selected VOC predictors; c) pattern of VOCs used as predictor variables in MLR model as selected by Pareto chart; the order in the list reflects the greatest predictive contribution to the model (VOC name_X; X= B blood or U urine or HS human semen). Notation for numeric values: comma "," is the decimal separator (SI).

a)

Immotiles %	Multiple R	Multiple R^2	Adjusted R^2	SS Model	df Model	MS Model	SS Residual	df Residual	MS Residual	F	p
HS + B + U	0,998866	0,997733	0,992633	5368,16	9	596,462	12,196	4	3,0491	195,619100	0,000063
HS + B	0,978713	0,957878	0,908736	10680,34	14	762,882	469,657	12	39,1381	19,492040	0,000004
HS + U	0,982579	0,965462	0,907900	6761,08	10	676,108	241,865	6	40,3108	16,772360	0,001299
B + U	0,997490	0,994986	0,982953	9214,68	12	767,890	46,4337	5	9,28673	82,68676	0,000062
HS	/	/	/	/	/	/	/	/	/	/	/
B	0,942534	0,888370	0,798003	17669,45	17	1039,379	2220,296	21	105,7284	9,830657	0,000002
U	0,989445	0,979000	0,923002	7929,90	16	495,619	170,096	6	28,3494	17,482550	0,000995

b)

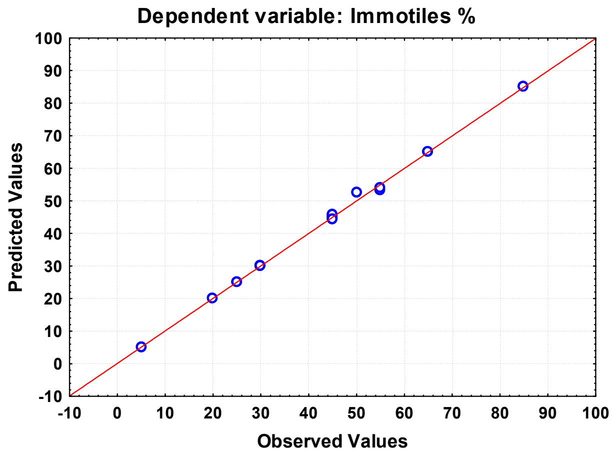

c) VOC predictors

1) 1-Anthracenamine_HS
2) 11H-Dibenzo[b,e][1,4]diazepin-11-one, 5,10-dihydro-5-[3-(methylamino)propyl]-_B
3) Fluoren-9-ol, 3,6-dimethoxy-9-(2-phenylethynyl)-_U
4) 1H-Indole, 5-methyl-2-phenyl-_U
5) Acetic acid, sodium salt_B
6) 2-Chloro-4-(4-methoxyphenyl)-6-(4-nitrophenyl)pyrimidine_B
7) n-Hexane_HS
8) Butanal, 2-methyl-_HS
9) Butanal_U

Figure S10 a) Overall fit of the MLR model described by the test of SS Whole Model vs. SS Residual for **sperm immotiles** (in percentage); b) Observed vs. Predicted values for sperm immotiles as result of the MLR analysis for the data group HS+B+U based on selected VOC predictors; c) pattern of VOCs used as predictor variables in MLR model as selected by Pareto chart; the order in the list reflects the greatest predictive contribution to the model (VOC name_X; X= B blood or U urine or HS human semen). Notation for numeric values: comma “,” is the decimal separator (SI).

a)

Morphology Anomalies %	Multiple R	Multiple R^2	Adjusted R^2	SS Model	df Model	MS Model	SS Residual	df Residual	MS Residual	F	p
HS + B + U	0,951849	0,906016	0,847275	137,26	5	27,452	14,239	8	1,7798	15,424110	0,000626
HS + B	0,892523	0,796597	0,669470	208,24	10	20,824	53,171	16	3,3232	6,266150	0,000656
HS + U	0,859512	0,738761	0,651681	113,86	4	28,464	40,262	12	3,3551	8,483729	0,001727
B + U	0,977555	0,955614	0,892205	186,93	10	18,693	8,6824	7	1,24035	15,07068	0,000805
HS	0,732860	0,537084	0,430257	144,459	6	24,077	124,51	26	4,7889	5,027608	0,001534
B	0,662527	0,438943	0,312252	197,28	7	28,182	252,159	31	8,1342	3,464687	0,007413
U	0,957156	0,916148	0,846271	182,91	10	18,291	16,741	12	1,3951	13,110940	0,000054

b)

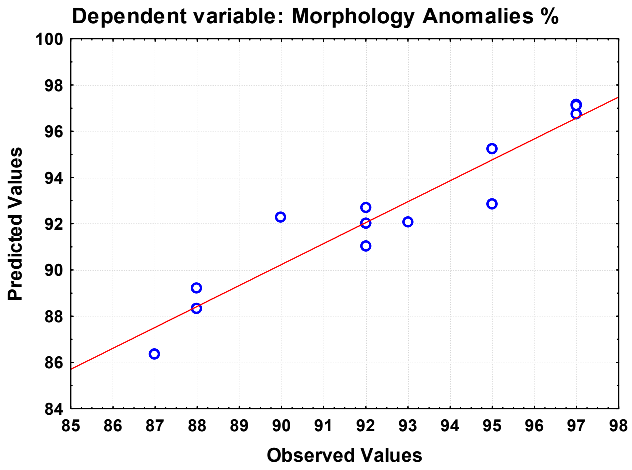

c) VOC predictors

1) Propane, 2-(ethenyloxy)-_U
2) n-Hexane_HS
3) Propane, 2-(ethenyloxy)-_B
4) 2-Pentanone_U
5) 3-Hexanone_U

Figure S11 a) Overall fit of the MLR model described by the test of SS Whole Model vs. SS Residual for sperm **morphology anomalies** (in percentage); b) Observed vs. Predicted values for sperm morphology anomalies as result of the MLR analysis for the data group HS+B+U based on selected VOC predictors; c) pattern of VOCs used as predictor variables in MLR model as selected by Pareto chart; the order in the list reflects the greatest predictive contribution to the model (VOC name_X; X= B blood or U urine or HS human semen). Notation for numeric values: comma "," is the decimal separator (SI).

a)

Head Anomalies%	Multiple R	Multiple R^2	Adjusted R^2	SS Model	df Model	MS Model	SS Residual	df Residual	MS Residual	F	p
HS + B + U	0,986646	0,973470	0,950730	230,92	6	38,487	6,293	7	0,8990	42,808650	0,000036
HS + B	0,999603	0,999206	0,994842	743,71	22	33,805	0,591	4	0,1477	228,939800	0,000041
HS + U	0,954244	0,910582	0,841034	218,43	7	31,205	21,450	9	2,3833	13,092920	0,000458
B + U	0,950425	0,903307	0,794527	298,94	9	33,216	32,0000	8	4,00000	8,30401	0,003320
HS	0,869429	0,755907	0,609451	622,501	12	51,875	201,01	20	10,0507	5,161331	0,000648
B	0,730072	0,533006	0,388076	726,80	9	80,756	636,789	29	21,9582	3,677693	0,003573
U	0,995314	0,990650	0,965718	342,68	16	21,417	3,234	6	0,5390	39,733730	0,000093

b)

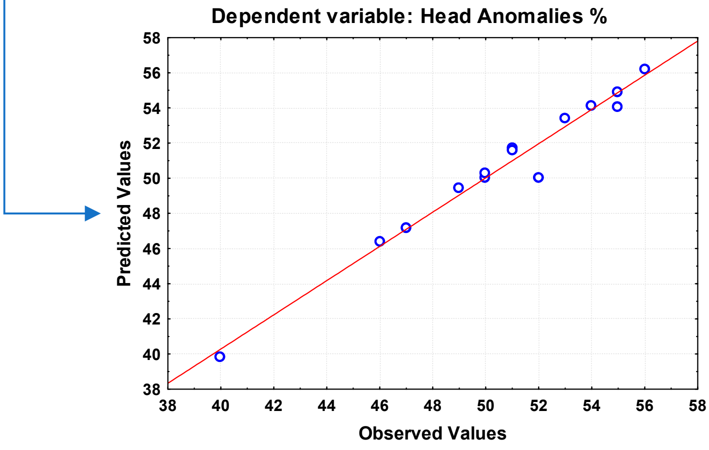

c) VOC predictors

1) 3-Hexanone_U
2) 4-(4-Chlorophenyl)-2,6-diphenylpyridine_U
3) Cyclohexane_B
4) Auramine_HS
5) Pentane_B
6) Hexanal_HS

Figure S12 a) Overall fit of the MLR model described by the test of SS Whole Model vs. SS Residual for sperm **head anomalies** (in percentage); b) Observed vs. Predicted values for sperm head anomalies as result of the MLR analysis for the data group HS+B+U based on selected VOC predictors; c) pattern of VOCs used as predictor variables in MLR model as selected by Pareto chart; the order in the list reflects the greatest predictive contribution to the model (VOC name_X; X= B blood or U urine or HS human semen). Notation for numeric values: comma "," is the decimal separator (SI).

a)

Neck Anomalies %	Multiple R	Multiple R^2	Adjusted R^2	SS Model	df Model	MS Model	SS Residual	df Residual	MS Residual	F	p
HS + B + U	0,997846	0,995696	0,988809	130,65	8	16,331	0,565	5	0,1130	144,578500	0,000017
HS + B	0,907557	0,823660	0,617931	208,36	14	14,883	44,607	12	3,7173	4,003611	0,010501
HS + U	0,902950	0,815320	0,769149	110,68	3	36,893	25,070	12	2,0892	17,659040	0,000107
B + U	0,813958	0,662528	0,478452	98,35	6	16,391	50,0959	11	4,55417	3,59921	0,031759
HS	0,761019	0,579150	0,461312	147,525	7	21,075	107,20	25	4,2881	4,914799	0,001346
B	0,721634	0,520755	0,299565	414,47	12	34,539	381,430	26	14,6704	2,354334	0,032890
U	0,985085	0,970392	0,918578	151,30	14	10,807	4,616	8	0,5770	18,728430	0,000140

b)

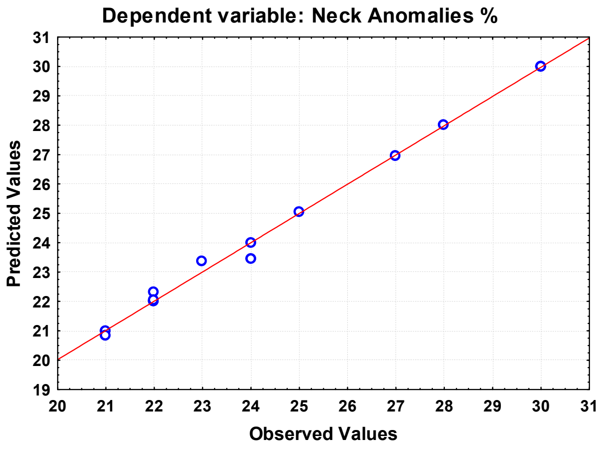

c) VOC predictors

1) Acetic acid, sodium salt_HS
2) Butanal, 2-methyl-_HS
3) 4-Heptanone_U
4) Butanal_U
5) .alpha.-Pinene_B
6) Disulfide, dimethyl_U
7) 2,4,5-Trioxoimidazolidine_U
8) 2-Ethyl-oxetane_B

Figure S13 a) Overall fit of the MLR model described by the test of SS Whole Model vs. SS Residual for sperm **neck anomalies** (in percentage); b) Observed vs. Predicted values for sperm neck anomalies as result of the MLR analysis for the data group HS+B+U based on selected VOC predictors; c) pattern of VOCs used as predictor variables in MLR model as selected by Pareto chart; the order in the list reflects the greatest predictive contribution to the model (VOC name_X; X= B blood or U urine or HS human semen). Notation for numeric values: comma "," is the decimal separator (SI).

a)

Tail Anomalies %	Multiple R	Multiple R^2	Adjusted R^2	SS Model	df Model	MS Model	SS Residual	df Residual	MS Residual	F	p
HS + B + U	0,999462	0,998925	0,993012	4331,05	11	393,732	4,661	2	2,3307	168,935000	0,005899
HS + B	0,999772	0,999545	0,996056	496,44	23	21,584	0,226	3	0,0753	286,495900	0,000293
HS + U	0,608807	0,370646	0,280738	106,70	2	53,351	181,180	14	12,9414	4,122518	0,039108
B + U	0,946014	0,894943	0,821404	275,64	7	39,378	32,3574	10	3,23574	12,16954	0,000358
HS	0,862206	0,743399	0,567830	411,077	13	31,621	141,89	19	7,4680	4,234225	0,002315
B	0,910540	0,829082	0,740205	682,82	13	52,525	140,766	25	5,6306	9,328398	0,000001
U	0,629402	0,396146	0,261957	126,46	4	31,614	192,761	18	10,7089	2,952139	0,048737

b)

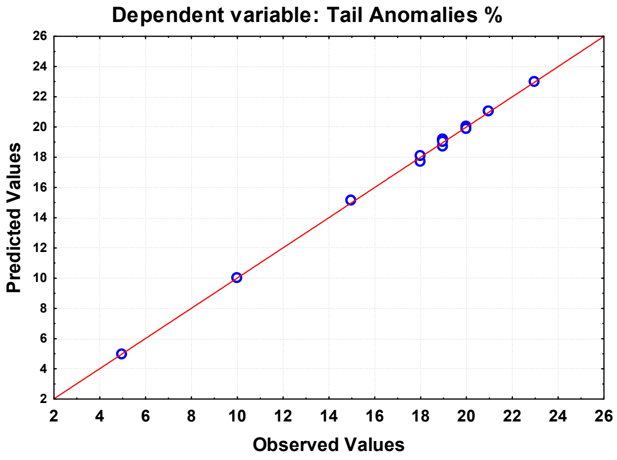

c) VOC predictors

1) n-Hexane_U
2) Butanal_HS
3) .alpha.-Pinene_B
4) Pentanal_HS
5) Acetic acid, sodium salt_HS
6) Propane,2-(ethenyloxy)-_B
7) 2-ethyl-oxetane_B
8) 11H-Dibenzo[b,e][1,4]diazepin-11-one, 5,10-dihydro-5-[3-(methylamino)propyl]-_U
9) Oxime,methoxy-phenyl-_HS

Figure S14 a) Overall fit of the MLR model described by the test of SS Whole Model vs. SS Residual for **sperm tail anomalies** (in percentage); b) Observed vs. Predicted values for sperm tail anomalies as result of the MLR analysis for the data group HS+B+U based on selected VOC predictors; c) pattern of VOCs used as predictor variables in MLR model as selected by Pareto chart; the order in the list reflects the greatest predictive contribution to the model (VOC name_X; X= B blood or U urine or HS human semen). Notation for numeric values: comma "," is the decimal separator (SI).

a)

Round cells 10^6/ml	Multiple R	Multiple R^2	Adjusted R^2	SS Model	df Model	MS Model	SS Residual	df Residual	MS Residual	F	p
HS + B + U	0,975114	0,950847	0,872202	142,15	8	17,769	7,348	5	1,4697	12,090350	0,006950
HS + B	0,918359	0,843384	0,728532	162,86	11	14,805	30,242	15	2,0162	7,343238	0,000306
HS + U	0,988538	0,977206	0,947901	192,57	9	21,396	4,492	7	0,6417	33,345000	0,000063
B + U	0,960418	0,922403	0,835106	192,12	9	21,346	16,1618	8	2,02022	10,56628	0,001458
HS	0,895012	0,801047	0,681675	202,624	12	16,885	50,33	20	2,5163	6,710513	0,000108
B	0,796776	0,634852	0,466323	174,97	12	14,581	100,638	26	3,8707	3,767008	0,002249
U	0,997482	0,994971	0,972338	272,45	18	15,136	1,377	4	0,3443	43,961700	0,001108

b)

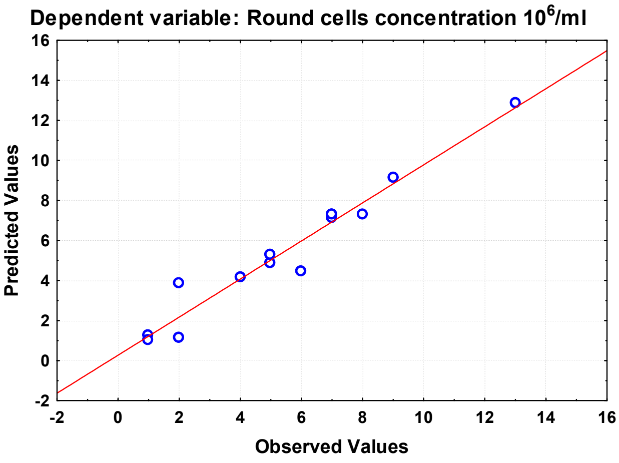

c) VOC predictors

1) 2H-Pyrrol-2-one, 1,5-dihydro-1-(4-methoxyphenyl)-5,5-diphenyl-_U
2) Octane_B
3) Acetone_HS
4) n-Hexane_HS
4) Pentanal_HS
5) Propane, 2-(ethenyloxy)-_U
6) 1-(6-Methyl-benzothiazol-2-yl)-3-(4-methyl-benzoyl)-thiourea_B
7) 3-Aminopyrrolidine_B

Figure S15 a) Overall fit of the MLR model described by the test of SS Whole Model vs. SS Residual for **round cells** (in percentage); b) Observed vs. Predicted values for round cells as result of the MLR analysis for the data group HS+B+U based on selected VOC predictors; c) pattern of VOCs used as predictor variables in MLR model as selected by Pareto chart; the order in the list reflects the greatest predictive contribution to the model (VOC name_X; X= B blood or U urine or HS human semen). Notation for numeric values: comma “,” is the decimal separator (SI).
